# Supplementary material for: The heat shock protein LarA activates the Lon protease in response to proteotoxic stress
Source: Nat Commun. 2023 Nov 22;14:7636. doi: 10.1038/s41467-023-43385-x (PMC10665427; doi:10.1038/s41467-023-43385-x)
Supplement: Supplementary file 1 — Supplementary Information [file 41467_2023_43385_MOESM1_ESM.pdf]

Supplementary Information

The heat shock protein LarA activates the Lon protease in response to proteotoxic stress

Deike J. Omnus[1*], Matthias J. Fink[1*], Aswathy Kallazhi[1], Maria Xandri Zaragoza[1], Axel Leppert[2], Michael Landreh[2,3] and Kristina Jonas[1#]

[1] Science for Life Laboratory and Department of Molecular Biosciences, The Wenner-Gren Institute, Stockholm University, Svante Arrhenius väg 20C, Stockholm 10691, Sweden

[2] Department of Microbiology, Tumor and Cell Biology, Karolinska Institutet, Solnavägen 9, 17165 Solna, Sweden.

[3] Department of Cell- and Molecular Biology, Uppsala University, Box 596, 751 24 Uppsala, Sweden.

*contributed equally

#Correspondence: kristina.jonas@su.se, Phone: +46 8 162580

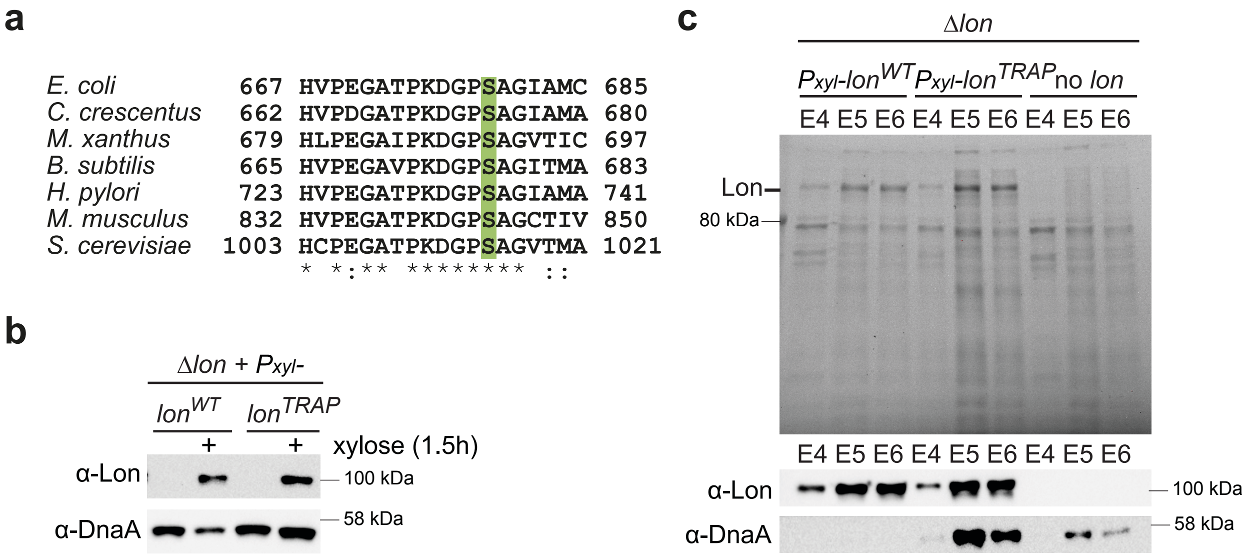

Supplementary Figure 1. A trapping approach allows co-purification of Lon-bound proteins.

(a) Alignment of indicated amino acid residue sequences of Lon from different species (*Escherichia coli* [AJF45020.1], *Caulobacter crescentus* [WP_004615160.1], *Myxococcus xanthus* [WP_201424642.1], *Bacillus subtilis* [ASZ62291.1], *Helicobacter pylori* [GHQ92543.1], *Mus musculus* [NP_083058.2], *Saccharomyces cerevisiae* [GHM90547.1]) illustrating the conservation of the catalytic serine residue (highlighted in green) of the peptidase domain.

(b) Immunoblot analysis confirming the expression Lon^{WT}-Twin-Strep-tag and Lon^{TRAP}-Twin-Strep-tag in Δ*lon* cells for the protease trapping experiment after 1.5 hours of xylose-induction (upper panel). Levels of the known Lon substrate DnaA are negatively affected by expression of Lon^{WT}-Twin-Strep-tag, but not of Lon^{TRAP}-Twin-Strep-tag, confirming its catalytic inactivity (lower panel).

(c) Stain-free SDS-PAGE gel analysis (upper panel, the bands corresponding to Lon derivatives are indicated at their height of migration) and immunoblot analysis (lower panel) of elution fractions 4-6 of the Twin-Strep-tag purification from strains described in Fig. 1a, confirming the purification of Lon constructs and co-purification of DnaA specifically in Lon^{TRAP}-Twin-Strep-tag eluates. Representative of the two replicates. Representative data from two independent experiments are shown.

Source data are provided as a Source Data file.

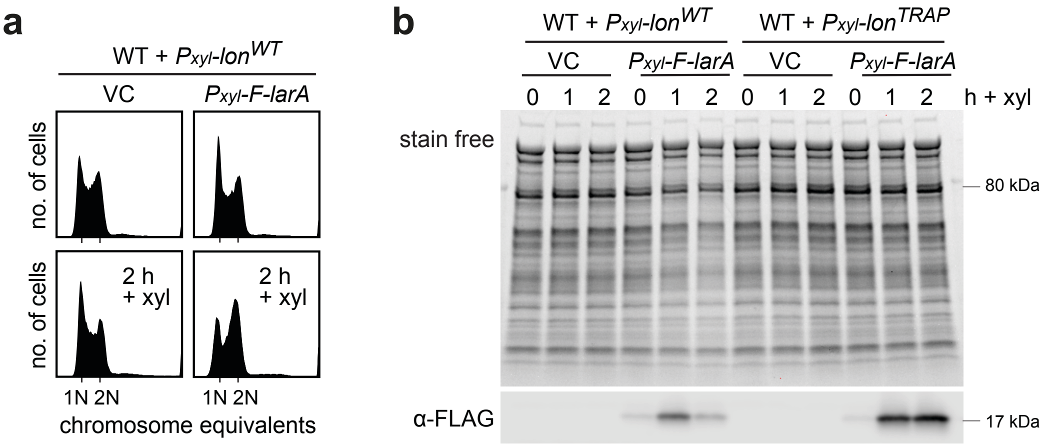

Supplementary Figure 2. Co-overexpression of *lon* and *larA* results in cell cycle arrest and reduced total protein content.

(a) Flow cytometry analysis of wild type cells (WT) with P_{xyl}-*Lon*^{WT}*-Twin-Strep-tag* integrated on the chromosome and harboring an empty vector (VC) or a plasmid carrying P_{xyl}*-3xFLAG-larA* (P_{xyl}*-F-larA*). Samples were taken just before (0 h) and 2 hours after xylose addition to induce expression of *lon*^{WT}*-Twin-Strep-tag* and *F-larA* (2 h). Representative data from two independent experiments are shown.

(b) Stain free SDS-PAGE gel (upper panel) and immunoblot analysis (lower panel) of extracts from wild type cells (WT) with either chromosomally integrated P_{xyl}-*Lon*^{WT}*-Twin-Strep-tag* or P_{xyl}-*Lon*^{TRAP}*-Twin-Strep-tag* and harboring an empty vector (VC) or a plasmid carrying P_{xyl}*-3xFLAG-larA* (P_{xyl}*-F-larA*). Samples were taken at time point 0 as well as 1 and 2 hours after xylose addition to induce expression of *lon*^{WT}*-Twin-Strep-tag* or *lon*^{TRAP}*-Twin-Strep-tag* as well as of *F-larA* where indicated. Representative data from two independent experiments are shown.

Source data are provided as a Source Data file.

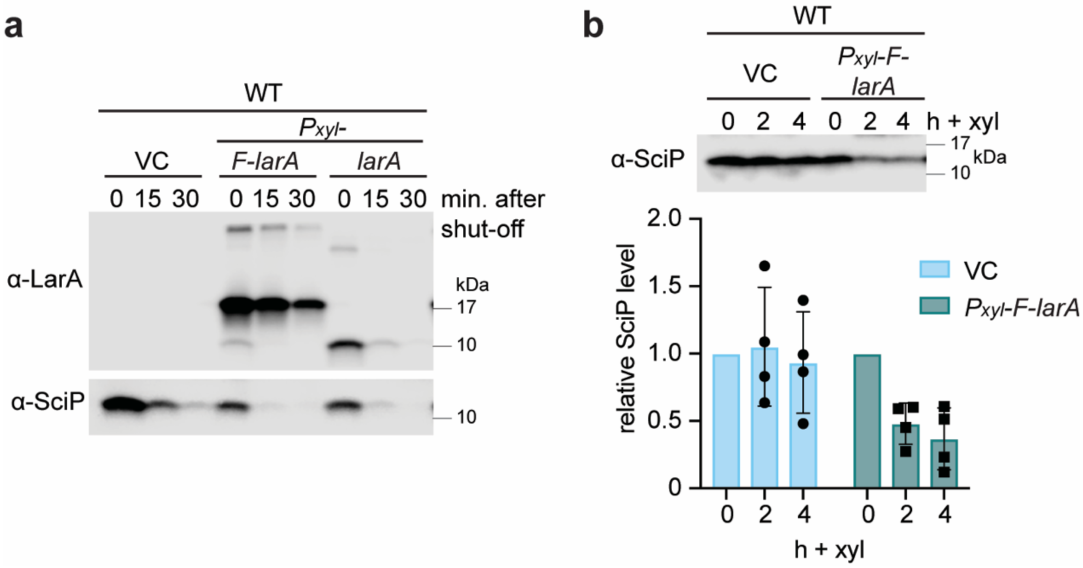

Supplementary Figure 3. LarA overexpression enhances SciP degradation and results in reduced SciP levels.

(a) Immunoblot analysis of LarA and SciP levels in wild type cells (WT) harboring an empty vector (VC) or plasmids carrying either P_{xyl}*-3xFLAG-larA* (P_{xyl}*-F-larA*) or P_{xyl}*-larA* (P_{xyl}*-larA*), respectively. Expression of LarA variants was induced by addition of xylose for 1.5 hour prior to addition of chloramphenicol to shut-off protein synthesis and samples to assess protein stability were subsequently taken at the indicated time points. Representative data are shown; n=2 biologically independent samples.

(b) Immunoblot analysis of SciP levels in wild type cells (WT) harboring an empty vector (VC) or a plasmid carrying P_{xyl}*-3xFLAG-larA* (P_{xyl}*-F-larA*), respectively. Samples were taken at the indicated time points after xylose addition to induce expression of *F-larA* where indicated. Quantifications show the mean values of relative SciP levels ± SD; n=4 biologically independent samples.

Source data are provided as a Source Data file.

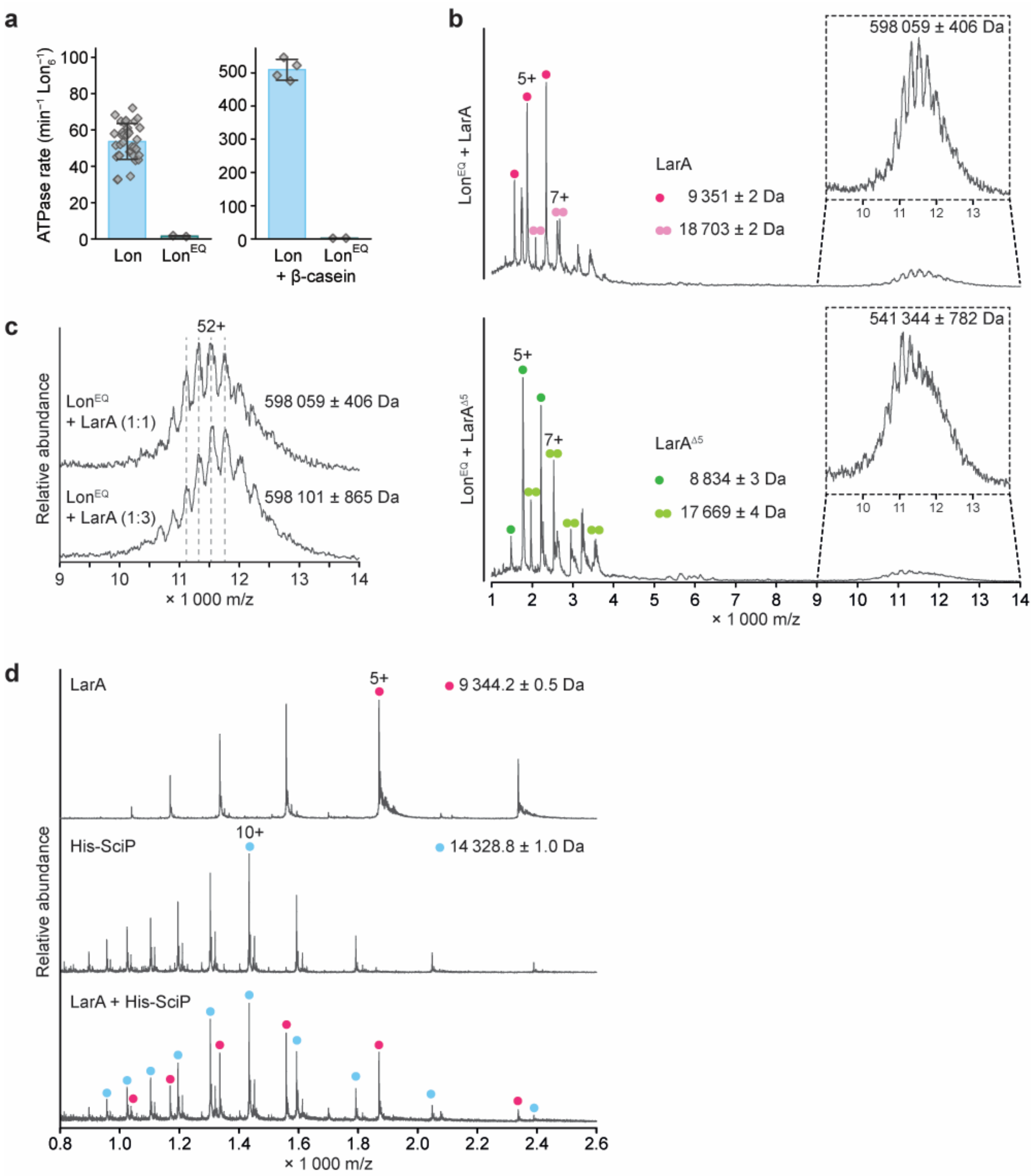

Supplementary Figure 4. Native MS data showing that LarA forms a complex with Lon, but not with SciP.

(a) ATPase rates of Lon and Lon^{EQ} in the absence or presence of 2 µM of the substrate β-casein (left and right panel, respectively). Bars represent the mean values ± SD; n=2 (Lon$^{EQ;}$ Lon^{EQ} + β-casein), 4 (Lon + β-casein) or 36 (Lon) independent measurements. Bars showing the ATPase rate of Lon without β-casein are reproduced from Fig. 3e.

(b) Full native mass spectra of Lon^{EQ} + LarA and Lon^{EQ} + LarA$^{\Delta5}$. LarA and LarA$^{\Delta5}$ were mainly detected as monomers (magenta and dark green, respectively) and to a low extent as dimers (light pink and light green, respectively). The experimentally determined masses of monomers and dimers are indicated. Inlays show the magnified *m/z* ranges where the complexes were detected.

The same regions are shown at higher magnification in Fig. 3. Representative data are shown; n=3 independent experiments.

(c) Spectra of LonEQ with equimolar and a 3-fold molar excess of LarA (1:1 and 1:3, respectively) with the experimentally determined masses. Representative data are shown; n=3 independent experiments.

(d) Spectra of LarA and His-SciP separately and after mixing (LarA + His-SciP) with the experimentally determined masses. In the spectrum recorded for an equimolar mixture of LarA and His-SciP, the peaks of LarA and His-SciP are marked by red and blue circles, respectively. Representative data are shown; n=2 independent experiments.

Source data are provided as a Source Data file.

a

```
Methylobacterium      ----MTVLNPSPLTPADLDPAEFQAADLAALGEGHIAYVRPIRSDEVRRFFPQA----PE
Bradyrhizobium        --------MSEGHVAFEYEAKNVSPETLATLGEGHIAYVKQIRSEDVPDLFPEA----PK
Agrobacterium         -------------MLLKEAHARLTQSQLAHIGEGEVGYIRKIRAEEVSRCFPEA----PD
Shinella              -------------MGLKSVTPTVSKADLAHLGAGEVGYIRKMKADEVSRVFPEA----PE
Bartonella            ---------------MGEHKQNLSLQDMTHSDTGQIAYLKKVCTDDLAKNFPDL----PP
Mesorhizobium         MALSGVLAPRRLEMTRTDENITMTSGEFAHLGEGSVAYLRKVSSDELLGRFPNI----GE
Brucella              ------------MEAKTMNKHVFTDNEFAHLGEGEIAYVRKIRSDDLARSFPSL----PP
Rhodobacterales       ---------------------MHTPFDFKDAGSRTV-YVKAVAVADLPKEVQAS------
Rhodobacterales       ---------------------METPYEFEEDGPDRIVYIRSVEVAELPEDLRDQ------
Rhodobacter           ---------------------MNTPFDFPE-GAGRLVYVRPVPVAELPEDVQSM------
Roseobacter           ---------------------MQTRYDFGADEGRPIVYVRPVHVADLPEDMREQ------
Oceanicaulis          ---------------------MDTETELMLDTTPTV-YVRTIDAAEIMEQAANA---GVK
Caulobacter           ------------MTPSTQHSRPLTSEAFAALGAPALVYVRPIKAAEILADAPEG-VEDLD
Phenylobacterium      -------------MEVVMMTPVLSTEAFAALGAPHLVYVRPVKAAEILASVPSSQIQGFE
Micavibrio            -----MTDCKTTDCKSASALRDISAQDFLGFGVQHVAYVRPA------------------
Magnetospirillum      ------------MQDLSTLLYPMSASDFASWGMPELAFVKRV------------------
Elioraea              --------MNTQKTRGQVSIPVPTRQQLAELGLGGLAYVRAVVVD---------------
Kiloniella            ----------------------MTEQDMTALGLEAVAYVKAVDVD---------------
                                                 :       : ::.

Methylobacterium      LTPGLDLFALLSASGAPIMLADSRDVVLANAAAHDLQPVSLH
Bradyrhizobium        IAPGLKLFALHAADGTPIMLTDSREAAVANAWSNELQAVSVH
Agrobacterium         LDPQLDLWALFGADGTPILLTDNRSSTFFKAAEDDLKTVSLH
Shinella              IDPSLDLWALFGADGTPILLTDNRSSTFYKAAEDDLRTVSLH
Bartonella            MTPGITVWALFGETGYPIILSDERSIALAGANEHELQIVTLH
Mesorhizobium         IAPGLELWALFAANGQPILLSDARDRALAGAMENDLTTVAIH
Brucella              IAPGLELWALFGASGEPIVLSDVRATALQGAHDHELRTVTLH
Rhodobacterales       AGGREQLYAVHDAEGGQLALVADRSLAFVLARQNDMTPVPVH
Rhodobacterales       AEGRDQIYAVHSEDGERLALVRDRAMAFALARSNDLAPVAVH
Rhodobacter           ARGLDHIYALHGADGERLALVRDRHLAFVLARQNDLAPVNVH
Roseobacter           AMGSTTLYALHSAEGERLALVRDRRLAFVLARQNNMNPVAVH
Oceanicaulis          LDSDATLYAIHDENGQRMAVFSDREAAFAAARDHGAEPVSVH
Caulobacter           LSPDQTLYAVCRADGERLAVLIDRDTAIAAALAHELAPVSVH
Phenylobacterium      LDPDQTLYAVHRADGERLAVLTDRESAVAAALAHELAPVSVH
Micavibrio            ILNGQKVFSIHAADGTPLGYHDNFDAALVLTRQNDLEPVRVQ
Magnetospirillum      VENSQVSWTIHSADGTAIGVAPSRDLAFAAIIQHELEPVSVH
Elioraea              ---GQSAFAIFGADGQQIGLAPDRALADAAIVQHEMVPVSVH
Kiloniella            ---GEACFGIFAADGTEITVVTNRDVAFAAVRQQDLEPLSVH
                             : :    *  :       .          : ::
```

b

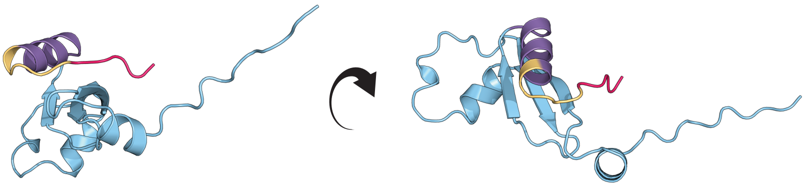

Supplementary Figure 5. The C-terminus of LarA is highly conserved and folds into a α-alpha-helix followed by a short unstructured region.

(a) Alignment of LarA amino acid residue sequences from different species illustrating the high level of conservation of the C-terminal 5 amino acids. *Caulobacter vibrioides* [YP_002519080.1], *Rhodobacter sphaeroides* [WP_002722317.1], *Brucella metlitensis* [WP_002968114.1], *Agrobacterium tumefaciens* [WP_006310223.1], *Bartonella Henselae* [WP_011180040.1], *Kiloniella laminariae* [WP_157230811.1], *Micavibrio aeruginosavorus* [WP_014101741.1], *Elioraea tepidiphila* [WP_019014739.1], *Magnetospirillum gryphiswaldense* [WP_024081410.1], Rhodobacterales bacterium [WP_008556561.1], *Methylobacterium radiotolerans* [WP_012320107.1], *Bradyrhizobium japonicum* [WP_018645064.1], *Shinella* sp. DD12 [WP_024270222.1], *Phenylobacterium zucineum* [WP_012520744.1], *Oceanicaulis* sp. HTCC2633 [WP_009802573.1], Rhodobacterales bacterium HTCC2150 [GenBank: EBA04284.1], *Roseobacter* sp. AzwK-3b [ZP_01904599.1], *Mesorhizobium* [WP_010911485.1]. Alignment was created using EMBL-EBI Multiple Sequence alignment MUSCLE [1].

(b) LarA structure predictions by Colabfold[2]. The C-terminal amino acid residues are predicted to form an α-helix (residues 71 to 81) followed by an unstructured region (residues 82 to 89). Together, those correspond to the C-terminal 20 amino acids analysed in this study. The amino acid residues that were removed in the LarA$^{\Delta 20}$, LarA$^{\Delta 10}$, LarA$^{\Delta 5}$ mutants are colored in magenta, yellow and purple (Δ20), yellow and red (Δ10) and red only (Δ5), respectively.

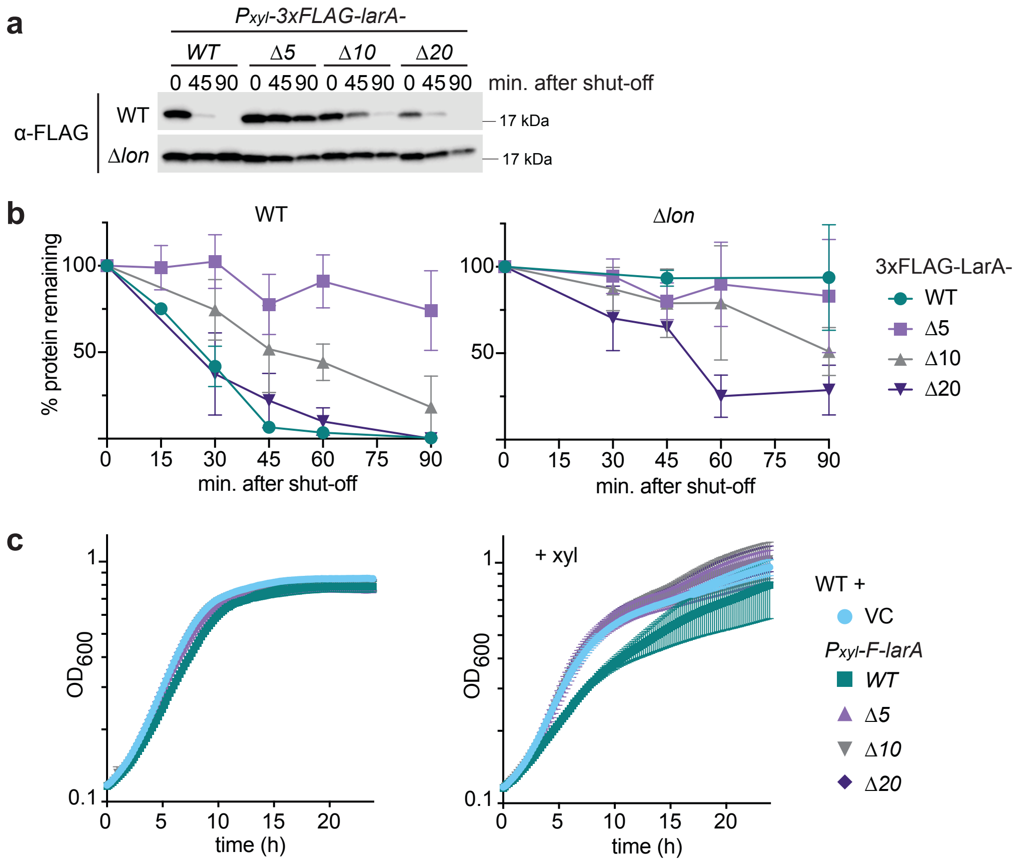

Supplementary Figure 6. The C-terminal amino acids of LarA are required for *in vivo* LarA degradation and LarA-dependent effects on growth.

(a) Immunoblot analysis of stability of 3xFLAG-LarA (F-LarA; WT) and F-LarA variants in wild type (upper panel) and Δ*lon* (lower panel) cells harboring plasmids carrying either *P_{xyl}-3xFLAG-larA* (*WT*), *P_{xyl}-3xFLAG-$larA^{\Delta 5}$* (Δ*5*), *P_{xyl}-3xFLAG-$larA^{\Delta 10}$* (Δ*10*), or *P_{xyl}-3xFLAG-$larA^{\Delta 20}$* (Δ*20*), respectively. Expression of F-LarA variants was induced by addition of xylose for 1 hour prior to addition of chloramphenicol to shut-off protein synthesis and samples to assess protein stability were subsequently taken at the indicated time points. Representative data are shown; n=2 biologically independent samples.

(b) Graphs show mean values ± SD of F-LarA variant levels in wild type (left hand side) and Δ*lon* cells (right hand side); n=2-6 biologically independent samples.

(c) Growth experiment using a plate reader to assess OD_{600} over 24 hours. Wild type cells (WT) harboring an empty vector (VC) or plasmids harboring either *P_{xyl}-3xFLAG-larA* (*WT*), *P_{xyl}-3xFLAG-$larA^{\Delta 5}$* (Δ*5*), *P_{xyl}-3xFLAG-$larA^{\Delta 10}$* (Δ*10*), or *P_{xyl}-3xFLAG-$larA^{\Delta 20}$* (Δ*20*), respectively, were grown under non-inducing conditions (left panel) or with xylose, i.e., *P_{xyl}*-inducing conditions (+ xyl; right panel). All growth curves display mean values ± SD; n=3 biologically independent samples from three independent experiments.

Source data are provided as a Source Data file.

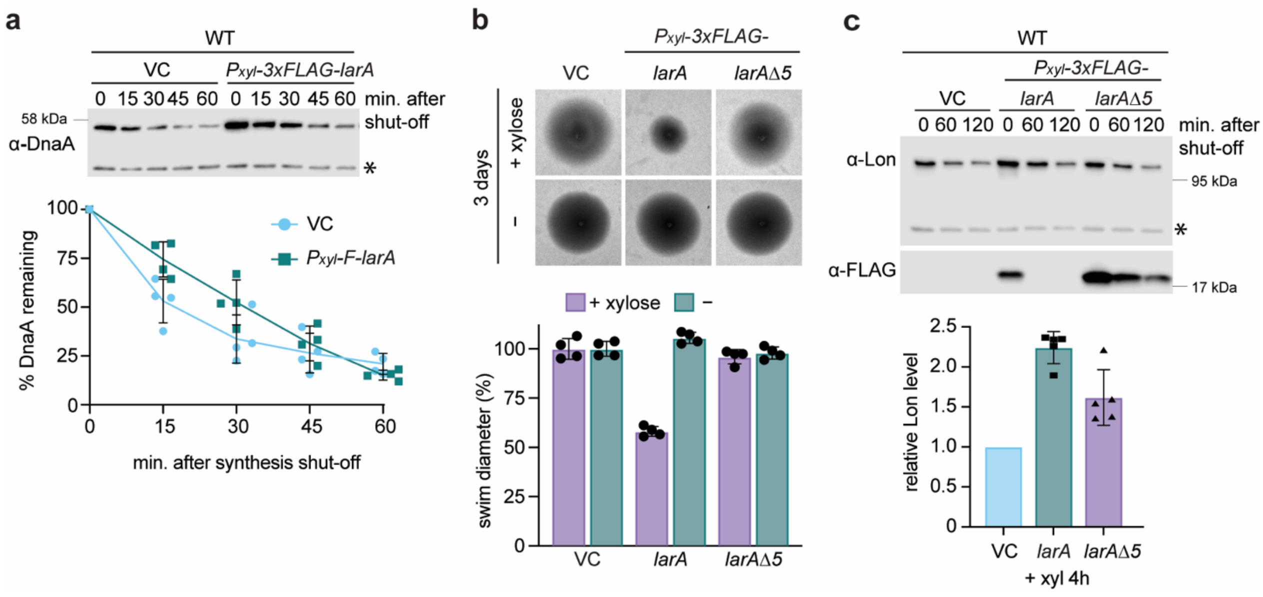

Supplementary Figure 7. Effects of *larA* overexpression on DnaA abundance and stability, soft-agar motility as well as Lon abundance.

(a) Immunoblot analysis of DnaA levels in wild type cells (WT) harboring an empty vector (VC) or a plasmid carrying P_{xyl}-*3xFLAG-larA* (P_{xyl}-*F-larA*). Expression of F-LarA was induced by addition of xylose for 1 hour prior to addition of chloramphenicol to shut-off protein synthesis and samples to assess DnaA stability were subsequently taken at the indicated time points. Graph shows means ± SD of DnaA levels; n=4 biologically independent samples.

(b) Motility assay in PYE soft agar containing gentamycin of wild type cells harboring the empty vector (VC) or overproducing 3xFLAG-tagged LarA (P_{xyl}-*3xFLAG-larA*) or F-LarA$^{\Delta5}$ (P_{xyl}-*3xFLAG-larA*$^{\Delta5}$), respectively, by xylose induction (+ xylose) in comparison to non-inducing conditions (−). The graph shows the relative swim diameters as means ± SD (means of VC were set to 100%); n=4 biologically independent samples.

(c) Immunoblot analysis of Lon levels (upper panel) in wild type cells (WT) harboring an empty vector (VC), a plasmid carrying P_{xyl}-*3xFLAG-larA* or P_{xyl}-*3xFLAG-larA*$^{\Delta5}$. Expression of *F-larA* and *F-larA*$^{\Delta5}$ was induced by addition of xylose for 4 hours prior to addition of chloramphenicol to shut-off protein synthesis and samples to assess Lon stability were subsequently taken at the indicated time points. Graph shows mean values ± SD of relative Lon steady state levels after 4 hours of xylose; n=5 biologically independent samples.

Source data are provided as a Source Data file.

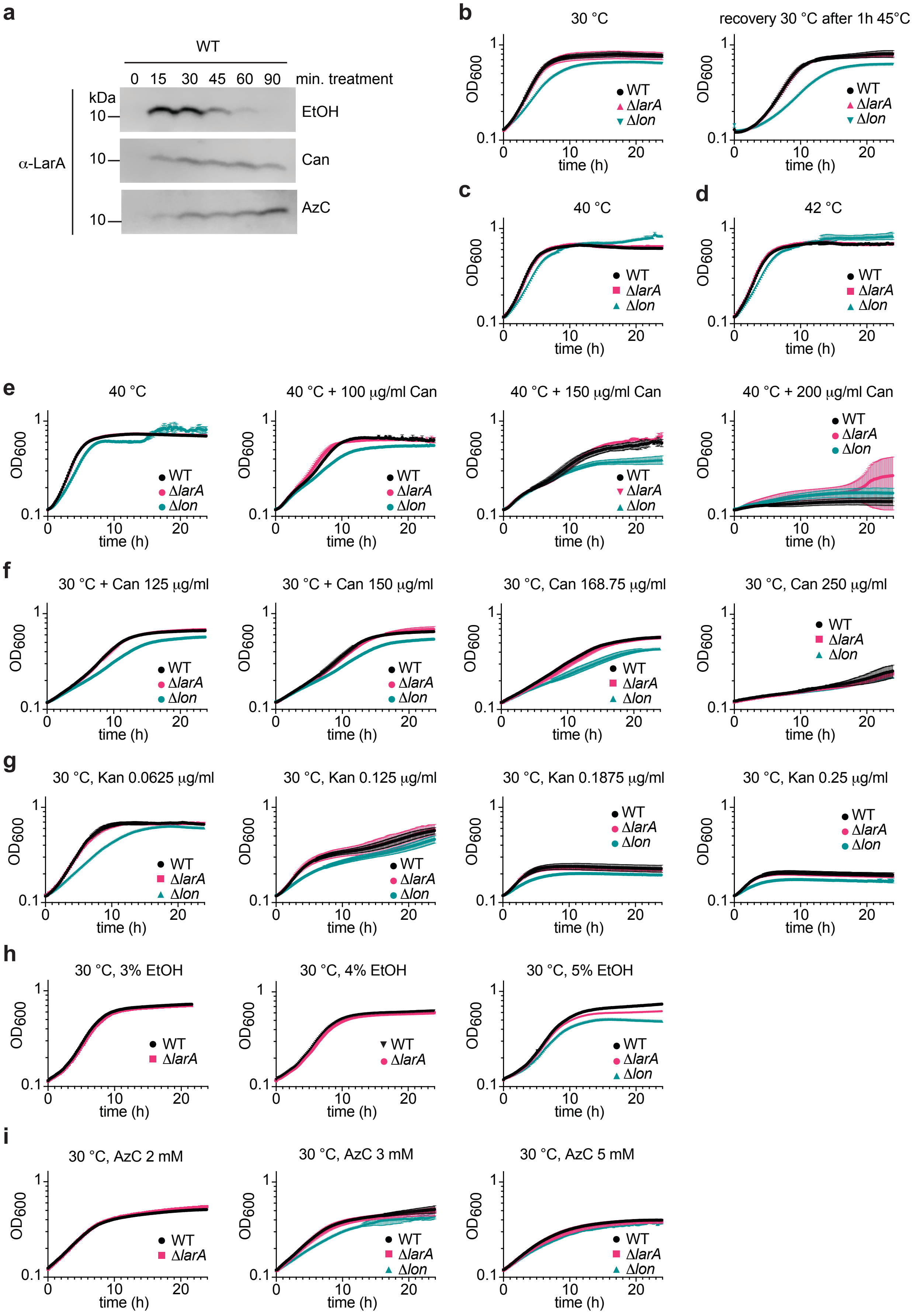

(Supplementary Figure 8 Legend on next page)

Supplementary Figure 8. Temporal pattern of LarA accumulation under distinct stress conditions and growth phenotypes of Δ*lon*, Δ*larA* and WT cells.

(a) Immunoblots showing induction of LarA levels in wild type (WT) cells before (0 min) and after treatment (15, 30, 45, 60 and 90 min) with ethanol (EtOH; 5 % final), L-canavanine (Can; 250 mg/ml final) or azetidine-2-carboxylate (AzC; 5 mM final). Representative data are shown; n=2 biologically independent samples.

(b) Growth experiment using a plate reader to assess OD_{600} over 24 hours. WT, Δ*larA* and Δ*lon* cells were grown at 30 °C, without previous treatment (left panel) or after a 1 h heat treatment at 45 °C. All growth curves display mean values ± SD; n=3 biologically independent cultures.

(c) Growth curves of wild type (WT), Δ*larA* and Δ*lon* cells at 40 °C. Data points represent the mean values ± SD; n=2 biologically independent cultures.

(d) Same as **(c)** but 42 °C.

(e) Same as **(c)** but in the presence of L-canavanine (Can; 100, 150 or 250 mg/ml final).

(f) Growth curves of WT, Δ*larA* and Δ*lon* cells at 30 °C in the presence of L-canavanine (Can; 125, 150, 168.75 or 250 mg/ml final). Data points represent mean values ± SD; n=2 or 3 biologically independent cultures; n=1 biologically independent culture for 250 mg/ml Δ*lon*.

(g) Growth curves of WT, Δ*larA* and Δ*lon* cells at 30 °C in the presence of kanamycin (Kan; 0.0625, 0.125, 0.1875 and 0.25 mg/ml final). Data points represent mean values ± SD; n=2 biologically independent cultures.

(h) Growth curves at 30 °C in the presence of ethanol (EtOH; 3, 4 and 5 % final). Data points represent mean values ± SD; n=1 (3% and 4% EtOH; 5% EtOH Δ*lon*) biologically independent culture; n=3 (5% EtOH Δ*larA*) and 2 (5% EtOH WT) biologically independent cultures.

(i) Growth curves at 30 °C in the presence of azetidine-2-carboxylate (AzC; 2, 3 and 5 mM final). Data points represent mean values ± SD; n=1 (2 mM; 5 mM Δ*lon*) biologically independent culture; n=3 (3 mM; 5 mM Δ*larA*) and n=2 (5 mM WT) biologically independent cultures.

Source data are provided as a Source Data file.

Supplementary Table 1. Kinetic parameters of LarA-activated His-SciP degradation.

	V_b (min^{-1} Lon$_6$$^{-1}$)	V_{max} (min^{-1} Lon$_6$$^{-1}$)	V_i (min^{-1} Lon$_6$$^{-1}$)	n	K_a (µM)	K_i (µM)
Value	1.5 ± 0.3	9.0 ± 3.0	6.8 ± 0.5	1.5 ± 0.5	0.3 ± 0.2	3.6 ± 5.9
p-value	2.01e-05 ***	0.00625 **	1.41e-11 ***	0.00901 **	0.04548 *	0.55446

Parameters show value ± standard error determined by fitting experimentally determined degradation rates of His-SciP at various LarA concentrations to equation (1). See Methods and Figure 4c for details and exact sample sizes. V_b: basal degradation without LarA; V_{max}: theoretical maximum degradation rate; V_i: max degradation at full inhibition; n: Hill constant; K_a: concentration with half-maximum activation; K_i: concentration with half-maximum inhibition. Asterisks indicate significance levels: *** $p < 0.001$, ** $p < 0.01$, * $p < 0.05$

Supplementary Table 2. Kinetic parameters of Lon-dependent His-SciP degradations in the presence or absence of LarA.

**Substrates**	V_{max} (k_{deg}) (min^{-1} Lon$_6$$^{-1}$)	K_m (µM)	**Hill constant n**	**catalytic efficiency** (µM^{-1} min^{-1} Lon$_6$$^{-1}$)
His-SciP	7.6 ± 1.7 ***	9.3 ± 4.0 *	–	0.8
His-SciP + LarA	15.0 ± 0.8 ***	1.8 ± 0.2 ***	1.9 ± 0.4 ***	8.3

Values for V_{max}, K_m and n represent parameters ± standard error determined by fitting the Michaelis-Menten and Hill equations to experimentally determined degradation rates with at least 3 independent measurements per concentration. See Methods figure 4d for details and exact sample sizes. The catalytic efficiency was calculated based on the fitted parameters. The p-values of the fitted parameter are as followed: His-SciP: V_{max} p = 0.000246; K_m p = 0.029364; His-SciP + LarA: V_{max} p = 1.47e-14; K_m p = 1.31e-09; n p = 8.83e-05. Asterisks indicate significance levels of the fitted parameters: *** $p < 0.001$, ** $p < 0.01$, * $p < 0.05$

Supplementary Table 3. Strains used in this study.

***Caulobacter crescentus* strains**			
**Name**	**Genotype**	**Marker**	**Reference**
NA1000 (CB15N)	WT, synchronizable derivative of wild-type CB15		Provided by Michael Laub
LS2382	Δ*lon (*NA1000 *lon*::*Ω)*	spec^R	[4]
KJ546 (=KG329)	Δ*lon* (*lon*::Ω re-introduced into NA1000 by phage transduction)	spec^R	
KJ1066 (=DJO002)	Δ*lon* P_{xyl}-*lon*^{WT}-*TwinStrep-tag*	kan^R	This study
KJ1067 (=DJO003)	Δ*lon* P_{xyl}-*lon*^{TRAP}-*TwinStrep-tag*	kan^R	This study
KJ1068 (=DJO004)	WT P_{xyl}-*lon*^{WT}-*TwinStrep-tag*	kan^R	This study
KJ1069 (=DJO005)	WT P_{xyl}-*lon*^{TRAP}-*TwinStrep-tag*	kan^R	This study
KJ1070 (=DJO006)	Δ*larA(CCNA_03707)*	tet^R	This study
***Escherichia coli* strains**			
**Name**	**Genotype**	**Marker**	**Reference**
DH5α	General cloning strain		Invitrogen
BL21-SI/ pCodonPlus	Salt-inducible BL21(DE3) strain for protein expression	chlor^R	Provided by Claes Andréasson
BL21(DE3)/ pLysS	Protein expression strain	chlor^R	Lab collection
ER2566	Lon-deficient B strain for protein expression		Provided by Peter Chien (originally from NEB)

Supplementary Table 4. Plasmids used in this study.

Name	Description	Marker	Reference
pBX-MCS-2	High copy, xylose-inducible expression	kan^R	[5]
pBX-MCS-4	High copy, xylose-inducible expression	$gent^R$	[5]
pDJO26	pBX-MCS-2 containing *lon*^{WT}-*TwinStrep-tag*	kan^R	This study
pDJO40	pBX-MCS-2 containing *lon*^{TRAP}-*TwinStrep-tag*	kan^R	This study
pDJO145	pBX-3xFLAG-4	$gent^R$	[6]
pDJO305	pBX-MCS-4 containing P_{xyl}-*larA-3xFLAG*	$gent^R$	This study
pDJO307	pBX-MCS-4 containing P_{xyl}-*3xFLAG-larA*	$gent^R$	This study
pDJO374	pBX-MCS-4 containing P_{xyl}-*3xFLAG-larAΔ5*	$gent^R$	This study
pDJO377	pBX-MCS-4 containing P_{xyl}-*3xFLAG-larAΔ10*	$gent^R$	This study
pDJO380	pBX-MCS-4 containing P_{xyl}-*3xFLAG-larAΔ20*	$gent^R$	This study
pDJO451	pBX-MCS-4 containing P_{xyl}-*3xFLAG-larA-H89D*	$gent^R$	This study
pDJO460	pBX-MCS-4 containing P_{xyl}-*3xFLAG-larA-V88D-H89D*	$gent^R$	This study
pDJO461	pBX-MCS-4 containing P_{xyl}-*3xFLAG-larA-V86A-V88A*	$gent^R$	This study
pML1716-*lon* (KJ600)	pML1716 containing *lon*	$chlor^R$	[7]
pXCHYN-2	Plasmid to integrate at *xylX* locus	kan^R	[5]
pDJO67	pXCHYN-2 containing *lon*^{WT}-*TwinStrep-tag*	kan^R	This study
pDJO70	pXCHYN-2 containing *lon*^{TRAP}-*TwinStrep-tag*	kan^R	This study
pNPTS138	Integrating vector for two-step recombination	kan^R	Lab collection, provided by Michael Laub
pDJO404	pNPTS138-*UHR-tet-DHR(larA)*, generation of a *tet*^{R}-marked deletion of *larA*(*CCNA_03707)*	tet^R, kan^R	This study
pBAD33-ccLon	pBAD33 derived vector for L-arabinose induced wildtype *C. crescentus* Lon expression	$chlor^R$	Provided by Peter Chien
pBAD33-Lon6his	pBAD33 derived vector for L-arabidose induced Lon-6xHis expression	$chlor^R$	Provided by Peter Chien
pSUMO-YHRC	Plasmid for protein expression using P_{T7} with an N-terminal 6xHis-SUMO tag; RRID:Addgene_54336	kan^R	[8]
pHis-SciP	pET-6xHis-sciP	amp^R	[9]
pMF65-c5	pSUMO-YHRC containing *6xHis-SUMO-larA*	kan^R	This study
pMF89-c2	pSUMO-YHRC containing *6xHis-SUMO-larAΔ5* (Δ85-89)	kan^R	This study

Name	Description	Marker	Reference
pMF82	pSUMO-YHRC containing *6xHis-SUMO-larA-V86A-V88A*	kan^R	This study
pMF81-c2	pSUMO-YHRC containing *6xHis-SUMO-larA-H89D*	kan^R	This study
pMF88-c4	pSUMO-YHRC containing *6xHis-SUMO-larA-V88D-H89D*	kan^R	This study
pMF58-c4	pSUMO-YHRC containing *6xHis-SUMO-fliX*	kan^R	This study
pMF79-c5	pSUMO-YHRC containing *6xHis-SUMO-LonE420Q* (a mutation in the Walker B motive)	kan^R	This study
pSH21-6xHis-titinI27-β20	pSH21 containing coding sequence for N-terminal 6xHis tagged human titinI27 domain with β-galactosidase degron at the C-terminus (*6xHis-titinI27-β20*)	amp^R	[10]
pAK002	pSH21 containing *6xHis-titinI27-larA5*	amp^R	This study
pAK003	pSH21 containing *6xHis-titinI27-larA10*	amp^R	This study
pAK004	pSH21 containing *6xHis-titinI27-larA20*	amp^R	This study
pAK005	pSH21 containing *6xHis-titinI27-larA5-V88D-H89D*	amp^R	This study
pAK006	pSH21 containing *6xHis-titinI27-LarA5-V86A-V88A*	amp^R	This study
pAK007	pSH21 containing *6xHis-titinI27-LarA5-H89D*	amp^R	This study
pAK008	pSH21 containing *6xHis-titinI27*	amp^R	This study

Supplementary Table 5. Oligonucleotides used in this study.

Name	Sequence (5'-3')	Reference
OAK057	CGCGGATCCCTAGTGGACAGACACCGGACTAGTCC	This study
OAK058	GGACTAGTCCGGTGTCTGTCCACTAGGGATCCGCG	This study
OAK059	CGCGGATCCCTAGTGGACAGACACCGGCGCCAGCTCATGGG CACTAGTCC	This study
OAK060	GGACTAGTGCCCATGAGCTGGCGCCGGTGTCTGTCCACTAG GGATCCGCG	This study
OAK061	CGCGGATCCCTAGTGGACAGACACCGGCGCCAGCTCATGGG CGAGGGCGGCGGCGATCGCGGTGTCGCGGTCACTAGTCC	This study
OAK062	GGACTAGTGACCGCGACACCGCGATCGCCGCCGCCCTCGCC CATGAGCTGGCGCCGGTGTCTGTCCACTAGGGATCCGCG	This study
OAK077	CGCGGATCCCTAGTCGACAGACACCGGACTAGTCC	This study
OAK078	GGACTAGTCCGGTGTCTGTCGACTAGGGATCCGCG	This study
OAK079	CGCGGATCCCTAGTCGTCAGACACCGGACTAGTCC	This study
OAK080	GGACTAGTCCGGTGTCTGACGACTAGGGATCCGCG	This study
OAK081	CGCGGATCCCTAGTGGGCAGACGCCGGACTAGTCC	This study
OAK082	GGACTAGTCCGGCGTCTGCCCACTAGGGATCCGCG	This study
OAK085	CGCGGATCCCTACTACTACTACTACTAACTAGTCC	This study
OAK086	GGACTAGTTAGTAGTAGTAGTAGTAGGGATCCGCG	This study
oDJO13	ATGGTCGTCTCCCCAAAACTC	This study
oDJO15	AGCCCGGGGGATCCACTAGTTC	This study
oDJO16	GAGTTTTGGGGAGACGACCATATGTCCGAACTACGTACGCTT CCTG	This study
oDJO171	GCTCGAGTTTTGGGGAGACGACCATATGACGCCCAGCACCCA ACAC	This study
oDJO172	CACCGTCATGGTCTTTGTAGTCCATATGGTGGACAGACACCG GCGCCAG	This study
oDJO173	GACTACAAGGACGACGACGACAAGGGTACCATGACGCCCAG CACCCAACAC	This study
oDJO174	AGTGGATCCCCCGGGCTGCAGTTAGGTACCCTAGTGGACAGA CACCG	This study
oDJO18	GAACTGCGGGTGGCTCCAGCTAGCGTGCGTCAGCATGGCGT CGCTG	This study
oDJO182	AGTGGATCCCCCGGGCTGCAGTTAGGTACCCTACGCCAGCTC ATGGGCGAG	This study
oDJO183	AGTGGATCCCCCGGGCTGCAGTTAGGTACCCTAGAGGGCGG CGGCGATC	This study
oDJO184	AGTGGATCCCCCGGGCTGCAGTTAGGTACCCTAGATCAGGAC CGCGAG	This study
oDJO185	CAATTGAAGCCGGCTGGCGCCAAGCTTCGGTCTTCACGAACG AAGTCGC	This study
oDJO186	GTATAGGAACTTCATGAATTCGATATCAAGCTTATCGATACCG GGTGCTGGGCGTCATAGGACC	This study
oDJO187	GTTCCTATACTTTCTAGAGAATAGGAACTTCTTGAATTCCTGCA GGAGCTGGCGCCGGTGTCTGTCC	This study
oDJO188	CCTGTACATCCGGAGACGCGTCACGGCCGAAGCTAGCGAATT CCAGTCGCTGGAGCGCCAAGG	This study

Name	Sequence (5'-3')	Reference
oDJO193	CTCCTCTTGAACCGAC	This study
oDJO194	GAACAGCGTGTTTCG	This study
oDJO197	AGTGGATCCCCCGGGCTGCAGTTAGGTACCCTAGTCGACAGACACCG	This study
oDJO198	AGTGGATCCCCCGGGCTGCAGTTAGGTACCCTAGTCGTCAGACACCG	This study
oDJO199	AGTGGATCCCCCGGGCTGCAGTTAGGTACCCTAGTGGGCAGACGCCGG	This study
oDJO20	GAACTAGTGGATCCCCCGGGCTTTAGGCGCCTTTTTCGAACTGC	This study
oDJO21	TGGCTCCACGATCCACCTCCCGATCCACCTCCGGAACCTCCACCTTTCTCGAACTGCGGGTGGCTCCAGC	This study
oDJO22	CTAGAACTAGTGGATCCCCCGGGCTTTAGGCGCCTTTTTCGAACTGCGGGTGGCTCCACGATCCACCTCC	This study
oDJO23	CACGCCCAAGGATGGTCCGGCTGCAGGCATCGCCATGGCCTTGG	This study
oDJO24	CCAAGGCCATGGCGATGCCTGCAGCCGGACCATCCTTGGGCGTG	This study
oDJO25	GCGTAACGTTCGAATTCTCCGGAGCTCTTAGGCGCCTTTTTCGAACTGC	This study
OFS25	CGGTATCGATAAGCTTGATATCGAATTCATGAAGTTCCTATAC	Lab collection
OFS26	CTGCAGGAATTCAAGAAGTTCCTATTCTCTAGAAAGTATAGGAAC	Lab collection
OFS932	CTCGAGTTTTGGGGAGACGACCATATGTCCGAACTACGTACGCTTCCTGTC	Lab collection
oMJF106	GTGCGGCCGCAAGCTTGTCGACGGAGCTCGAATTCGGATCCTAATCGTCAGACACCGGCGCCAGCTC	This study
oMJF107	GTGCGGCCGCAAGCTTGTCGACGGAGCTCGAATTCGGATCCTACGCCAGCTCATGGGCGAG	This study
oMJF34	CCCACCAATCTGTTCTCTGTG	[6]
oMJF36	CATGCATCATCAGGAGTACGG	[6]
oMJF37	GATCCGAATTCGAGCTCC	[6]
oMJF38	GAATTTATGCCTCTTCCGACC	[6]
oMJF47	AGAGAACAGATTGGTGGGATGAAGGTTTCCAGCACG	This study
oMJF48	GGAGCTCGAATTCGGATCTGCTATCCGGCCCTG	This study
oMJF67	TAACGATATTATTGAGGCTCACAGAGAACAGATTGGTGGGATGACGCCCAGCACCCAACAC	This study
oMJF68	GTGCGGCCGCAAGCTTGTCGACGGAGCTCGAATTCGGATCCTAGTGGACAGACACCG	This study
oMJF90	TAACGATATTATTGAGGCTCACAGAGAACAGATTGGTGGGATGTCCGAACTACGTACGCTTCCTGTCTTGC	This study
oMJF91	GTGCGGCCGCAAGCTTGTCGACGGAGCTCGAATTCGGATCTTAGTGCGTCAGCATGGCGTCGCTGTC	This study
oMJF94	CCATCTTGTCGATCTGGTCCAGCAGGACGAAGG	This study
oMJF95	CCTTCGTCCTGCTGGACCAGATCGACAAGATGG	This study
oMJF96	GTGCGGCCGCAAGCTTGTCGACGGAGCTCGAATTCGGATCCTAATCGACAGACACCGGCGCCAGC	This study
oMJF97	GTGCGGCCGCAAGCTTGTCGACGGAGCTCGAATTCGGATCCTAGTGTGCAGATGCCGGCGCCAGCTCATGGGCGAG	This study

Name	Sequence (5'-3')	Reference
RecUni-1	ATGCCGTTTGTGATGGCTTCCATGTCG	[5]
RecXyl-2	TCTTCCGGCAGGAATTCACTCACGCC	[5]
T7	TAATACGACTCACTATAGGG	common primer
T7 terminator	GCTAGTTATTGCTCAGCGG	common primer

Supplementary Datasets (provided as separate Excel spreadsheets)

Supplementary Dataset 1. Proteomics-based identification of Lon-bound proteins.

Supplementary Dataset 2. Quantitative proteomics analysis of LarA overexpressing cells.

Supplementary Information References

1 Madeira, F. *et al.* The EMBL-EBI search and sequence analysis tools APIs in 2019. *Nucleic Acids Res* **47**, W636-W641, doi:10.1093/nar/gkz268 (2019).

2 Mirdita, M., Ovchinnikov, S., Steinegger, M. ColabFold: Making protein folding accessible to all. *Nature Methods* **19**, 679-682 (2022).

3 Walsh, R., Martin, E. & Darvesh, S. A versatile equation to describe reversible enzyme inhibition and activation kinetics: modeling beta-galactosidase and butyrylcholinesterase. *Biochim Biophys Acta* **1770**, 733-746, doi:10.1016/j.bbagen.2007.01.001 (2007).

4 Wright, R., Stephens, C., Zweiger, G., Shapiro, L. & Alley, M. R. *Caulobacter* Lon protease has a critical role in cell-cycle control of DNA methylation. *Genes & development* **10**, 1532-1542 (1996).

5 Thanbichler, M., Iniesta, A. A. & Shapiro, L. A comprehensive set of plasmids for vanillate- and xylose-inducible gene expression in *Caulobacter crescentus*. *Nucleic Acids Res* **35**, e137, doi:10.1093/nar/gkm818 (2007).

6 Omnus, D. J., Fink, M. J., Szwedo, K. & Jonas, K. The Lon protease temporally restricts polar cell differentiation events during the *Caulobacter* cell cycle. *Elife* **10**, doi:10.7554/eLife.73875 (2021).

7 Jonas, K., Liu, J., Chien, P. & Laub, M. T. Proteotoxic stress induces a cell-cycle arrest by stimulating Lon to degrade the replication initiator DnaA. *Cell* **154**, 623-636, doi:10.1016/j.cell.2013.06.034 (2013).

8 Holmberg, M. A., Gowda, N. K. & Andreasson, C. A versatile bacterial expression vector designed for single-step cloning of multiple DNA fragments using homologous recombination. *Protein Expr Purif* **98**, 38-45, doi:10.1016/j.pep.2014.03.002 (2014).

9 Gora, K. G. *et al.* A cell-type-specific protein-protein interaction modulates transcriptional activity of a master regulator in *Caulobacter crescentus*. *Molecular cell* **39**, 455-467, doi:10.1016/j.molcel.2010.06.024 (2010).

10 Wohlever, M. L., Nager, A. R., Baker, T. A. & Sauer, R. T. Engineering fluorescent protein substrates for the AAA+ Lon protease. *Protein Eng Des Sel* **26**, 299-305, doi:10.1093/protein/gzs105 (2013).
