# Supplementary material for: The heat shock protein LarA activates the Lon protease in response to proteotoxic stress
Source: Nat Commun. 2023 Nov 22;14:7636. doi: 10.1038/s41467-023-43385-x (PMC10665427; doi:10.1038/s41467-023-43385-x)
Supplement: Supplementary file 5 — Reporting Summary [file 41467_2023_43385_MOESM5_ESM.pdf]

nature portfolio

Corresponding author(s): Kristina Jonas

Last updated by author(s): Oct 16, 2023

Reporting Summary

Nature Portfolio wishes to improve the reproducibility of the work that we publish. This form provides structure for consistency and transparency in reporting. For further information on Nature Portfolio policies, see our Editorial Policies and the Editorial Policy Checklist.

Statistics

For all statistical analyses, confirm that the following items are present in the figure legend, table legend, main text, or Methods section.

n/a	Confirmed	
☐	☒	The exact sample size (*n*) for each experimental group/condition, given as a discrete number and unit of measurement
☐	☒	A statement on whether measurements were taken from distinct samples or whether the same sample was measured repeatedly
☐	☒	The statistical test(s) used AND whether they are one- or two-sided<br>*Only common tests should be described solely by name; describe more complex techniques in the Methods section.*
☒	☐	A description of all covariates tested
☐	☒	A description of any assumptions or corrections, such as tests of normality and adjustment for multiple comparisons
☐	☒	A full description of the statistical parameters including central tendency (e.g. means) or other basic estimates (e.g. regression coefficient) AND variation (e.g. standard deviation) or associated estimates of uncertainty (e.g. confidence intervals)
☐	☒	For null hypothesis testing, the test statistic (e.g. *F*, *t*, *r*) with confidence intervals, effect sizes, degrees of freedom and *P* value noted<br>*Give P values as exact values whenever suitable.*
☒	☐	For Bayesian analysis, information on the choice of priors and Markov chain Monte Carlo settings
☒	☐	For hierarchical and complex designs, identification of the appropriate level for tests and full reporting of outcomes
☒	☐	Estimates of effect sizes (e.g. Cohen's *d*, Pearson's *r*), indicating how they were calculated

Our web collection on statistics for biologists contains articles on many of the points above.

Software and code

Policy information about availability of computer code

Data collection	BioRad ImageLab 5.1;<br>LI-COR Image Studio 5.2.5;<br>TECAN SparkControl magellan 2.2<br>MassLynx 4.1
Data analysis	Microsoft Excel 2016;<br>BioRad ImageLab 5.1/6.1.0;<br>GraphPad Prism 9.4.1;<br>R v4.2.1/RStudio 2021.09.0;<br>github.com/Matthionine/DetermineATPaseRate (extraction of ATPase rates)

For manuscripts utilizing custom algorithms or software that are central to the research but not yet described in published literature, software must be made available to editors and reviewers. We strongly encourage code deposition in a community repository (e.g. GitHub). See the Nature Portfolio guidelines for submitting code & software for further information.

Data

Policy information about availability of data

All manuscripts must include a data availability statement. This statement should provide the following information, where applicable:

- Accession codes, unique identifiers, or web links for publicly available datasets
- A description of any restrictions on data availability
- For clinical datasets or third party data, please ensure that the statement adheres to our policy

All relevant data supporting the findings of this study are provided in the main figures and Supplementary Information files. The mass spectrometry proteomics data have been deposited to the ProteomeXchange Consortium via the PRIDE57 partner repository with the database identifier PXD036514 (https://proteomecentral.proteomexchange.org/cgi/GetDataset?ID=PXD036514) and will be made accessible upon publication. Until then the data can be accessed using the following login details:
Username: reviewer_pxd036514@ebi.ac.uk
Password: RDhG4EQj
Source data underlying Figures 1c-f, 2a-f, 3a-e, 4b-g, 5b-c, 6a-d, 7c-g and Supplementary Figures 1b-c, 2b, 3a-b, 4a, 6a-c, 7a-c and 8a-i are provided as Source Data files.

Human research participants

Policy information about studies involving human research participants and Sex and Gender in Research.

Reporting on sex and gender	no human research participants present in this study
Population characteristics	see above
Recruitment	see above
Ethics oversight	see above

Note that full information on the approval of the study protocol must also be provided in the manuscript.

Field-specific reporting

Please select the one below that is the best fit for your research. If you are not sure, read the appropriate sections before making your selection.

[x] Life sciences [ ] Behavioural & social sciences [ ] Ecological, evolutionary & environmental sciences

For a reference copy of the document with all sections, see nature.com/documents/nr-reporting-summary-flat.pdf

Life sciences study design

All studies must disclose on these points even when the disclosure is negative.

Sample size	Experiments were conducted in multiple replicates as stated in the figure legends. For most experiments we used a standard sample size of three. For some strains or experimental conditions, the sample size was higher (n=4, 5, 6 or higher), and a few data points are based on two replicates. Biologically independent samples were collected from cultures originating from different colonies of the same strain. Replicates of in vitro assays are based either on independent experiments or in some cases on independently prepared reactions. The sample size and type of replicate for each experiment are clearly stated in the figure legends.
Data exclusions	No data exclusions occurred.
Replication	All experiments were conducted multiple times (as stated in the figure legends). The results were reproducible. Furthermore, different experimental assays yielded consistent results.
Randomization	Randomization is not applicable for this study.
Blinding	Blinding is not applicable for this study.

Reporting for specific materials, systems and methods

We require information from authors about some types of materials, experimental systems and methods used in many studies. Here, indicate whether each material, system or method listed is relevant to your study. If you are not sure if a list item applies to your research, read the appropriate section before selecting a response.

Materials & experimental systems

n/a	Involved in the study
☐	☒ Antibodies
☒	☐ Eukaryotic cell lines
☒	☐ Palaeontology and archaeology
☒	☐ Animals and other organisms
☒	☐ Clinical data
☒	☐ Dual use research of concern

Methods

n/a	Involved in the study
☒	☐ ChIP-seq
☐	☒ Flow cytometry
☒	☐ MRI-based neuroimaging

Antibodies

Antibodies used

anti-DnaA, 1:5000, (Jonas et al., 2011)
anti-Lon, 1:10000, (Vieux et al., 2013)
anti-FLAG M2 antibody produced in mouse, 1:1 000, (Sigma, catalog number F1804-1MG, clone: clone M2, lot number: SLCF 4933)
anti-SciP, 1:2 000, (Gora et al., 2010)
anti-FliK-C, 1:300, (Omnus et al., 2021)
anti-LarA, 1:250, (this study)

Goat anti-Rabbit IgG (H+L) Secondary Antibody, HRP (Thermo Fisher Scientific, Invitrogen, catalog number: 32460, lot number: WG331983)
Goat anti-Mouse IgG (H+L) Secondary Antibody, HRP (Thermo Fisher Scientific, Invitrogen, catalog number: 32430, lot number: SG251810 and OE185747),

Validation

anti-DnaA, (Jonas et al., 2011);
anti-Lon, (Vieux et al., 2013);
anti-FLAG M2 antibody, (Sigma);
anti-SciP, (Gora et al., 2010);
anti-FliK-C, (Omnus et al., 2021);
anti-LarA, (this study, figure 7. To validate the anti-LarA antibody, it was tested on lysates from a ΔlarA strain and in contrast to the WT and Δlon cell lysates, no band was observed indicating specificity of the antibody to LarA (figure 7D))

Flow Cytometry

Plots

Confirm that:

☐ The axis labels state the marker and fluorochrome used (e.g. CD4-FITC).

☐ The axis scales are clearly visible. Include numbers along axes only for bottom left plot of group (a 'group' is an analysis of identical markers).

☐ All plots are contour plots with outliers or pseudocolor plots.

☐ A numerical value for number of cells or percentage (with statistics) is provided.

Methodology

Sample preparation — *Describe the sample preparation, detailing the biological source of the cells and any tissue processing steps used.*

Instrument — *Identify the instrument used for data collection, specifying make and model number.*

Software — *Describe the software used to collect and analyze the flow cytometry data. For custom code that has been deposited into a community repository, provide accession details.*

Cell population abundance — *Describe the abundance of the relevant cell populations within post-sort fractions, providing details on the purity of the samples and how it was determined.*

Gating strategy — *Describe the gating strategy used for all relevant experiments, specifying the preliminary FSC/SSC gates of the starting cell population, indicating where boundaries between "positive" and "negative" staining cell populations are defined.*

☐ Tick this box to confirm that a figure exemplifying the gating strategy is provided in the Supplementary Information.
