# Supplementary material for: The heat shock protein LarA activates the Lon protease in response to proteotoxic stress
Source: Nat Commun. 2023 Nov 22;14:7636. doi: 10.1038/s41467-023-43385-x (PMC10665427; doi:10.1038/s41467-023-43385-x)

Uncroppped Western Blot and Gel images with molecular weight markers

Figure 4b
anti-FLAG

uncropped

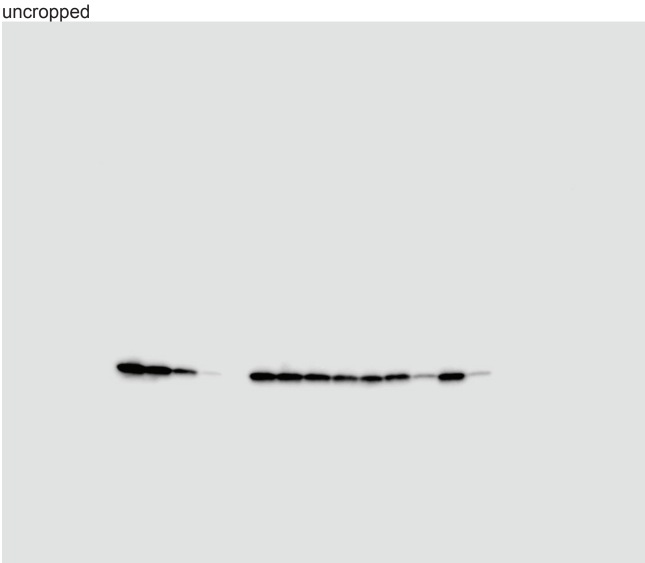

uncropped, overlayed with MWM image, with labelling

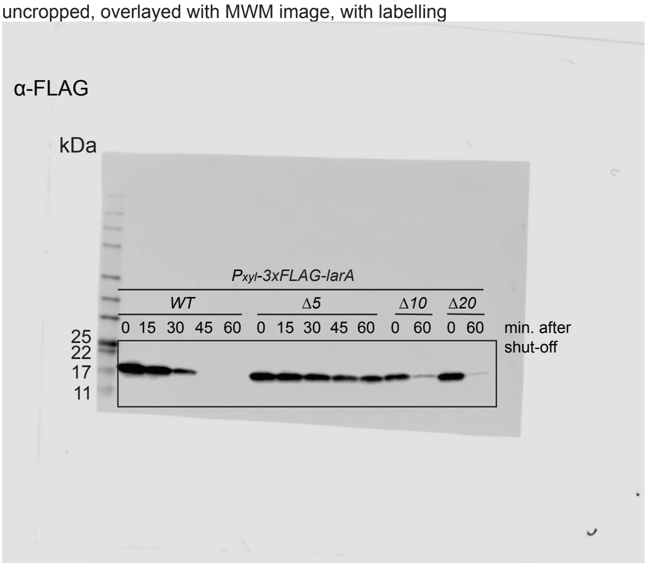

Figure 4b
anti-SciP
(inc. after
anti-M2)

uncropped

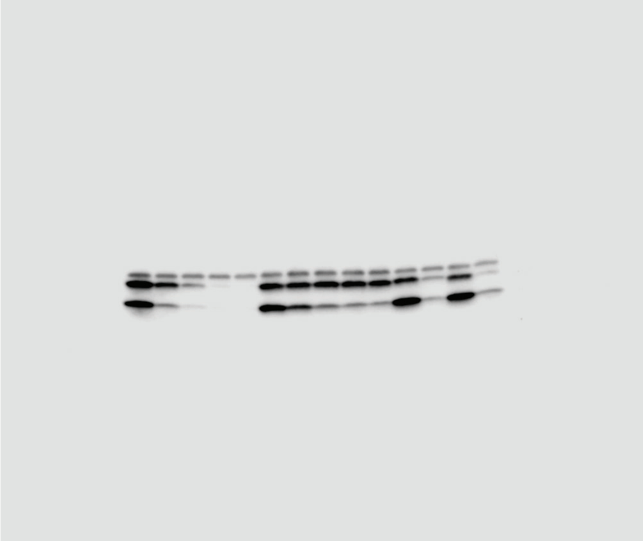

uncropped, overlayed with MWM image, with labelling

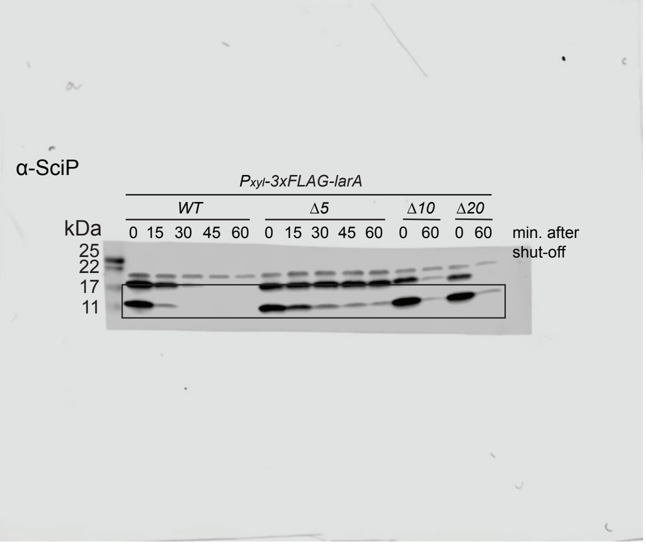

Figure 4c anti-FLAG, left panel

uncropped

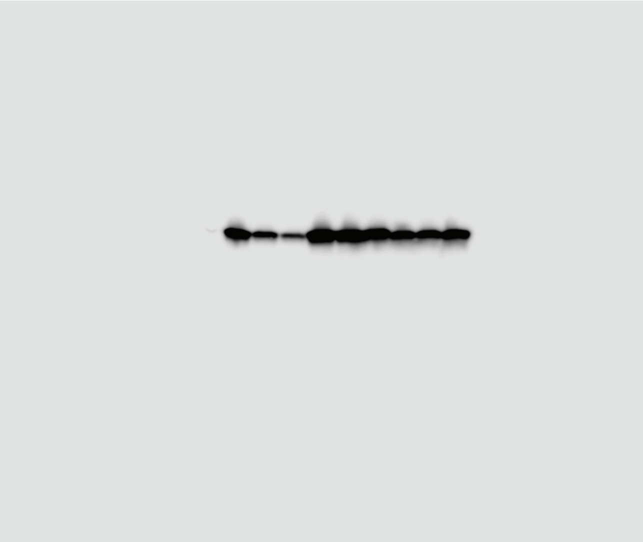

uncropped, overlayed with MWM image, with labelling

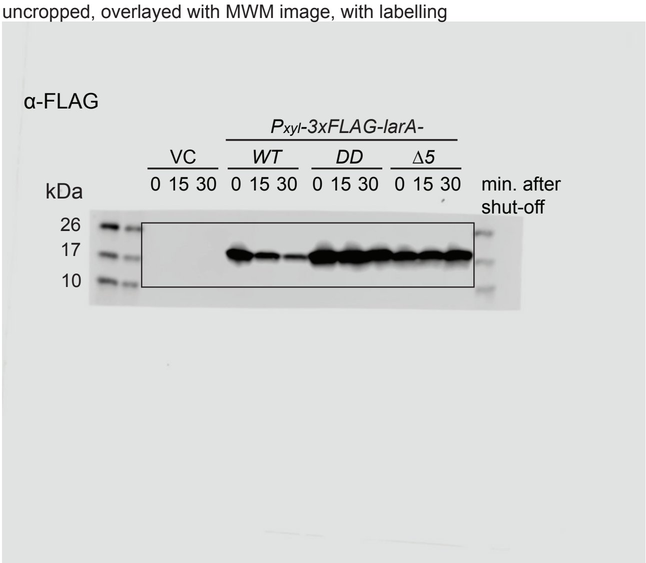

Figure 4c anti-FLAG, right panel

uncropped

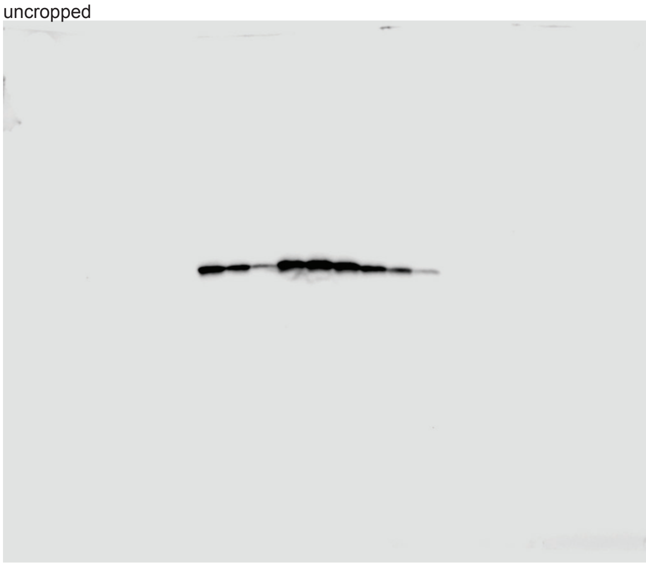

uncropped, overlayed with MWM image, with labelling

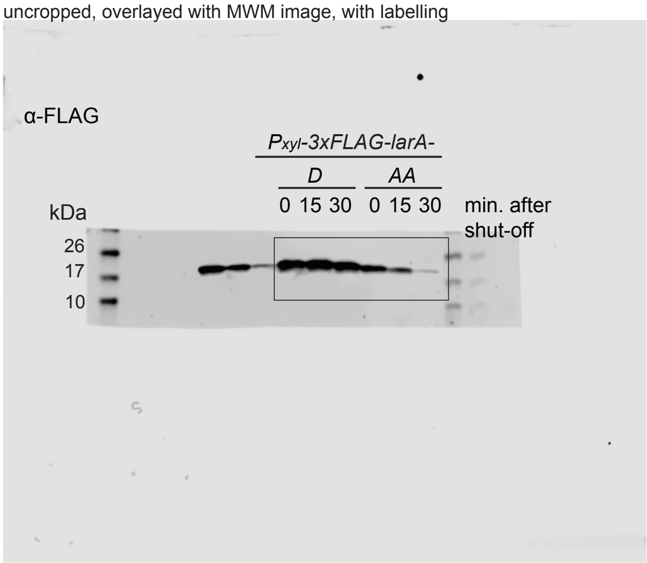

Figure 4c
anti-SciP,
left panel

uncropped

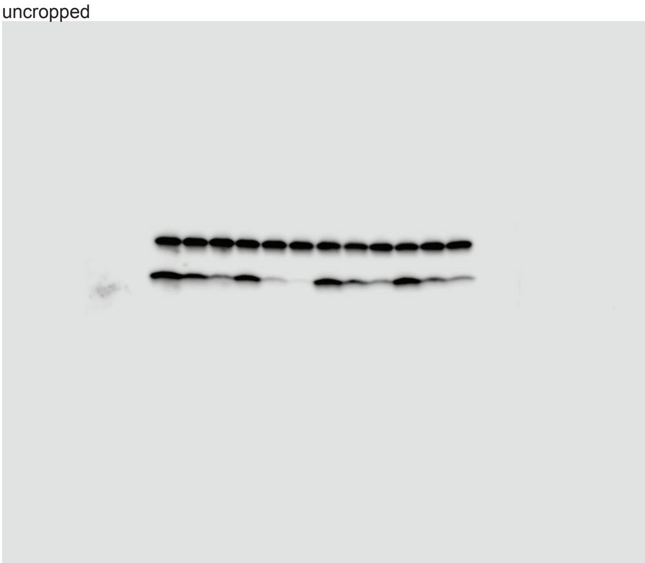

uncropped, overlayed with MWM image, with labelling

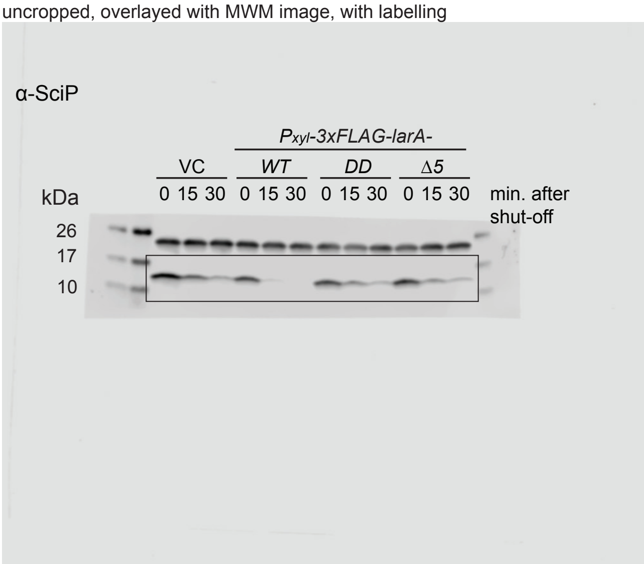

Figure 4c
anti-SciP,
right panel

uncropped

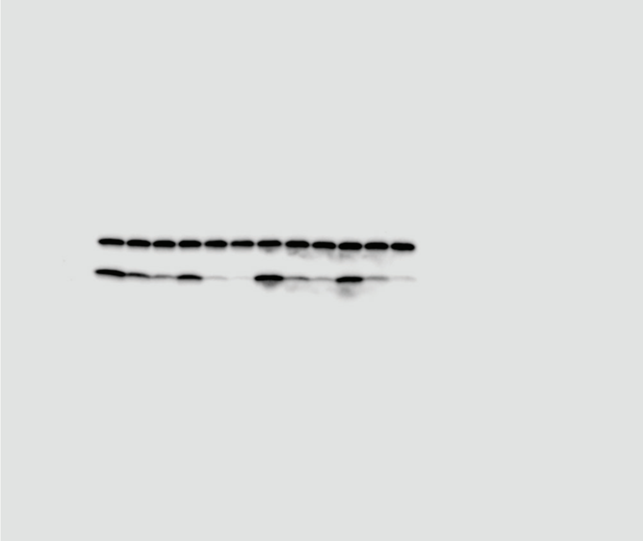

uncropped, overlayed with MWM image, with labelling

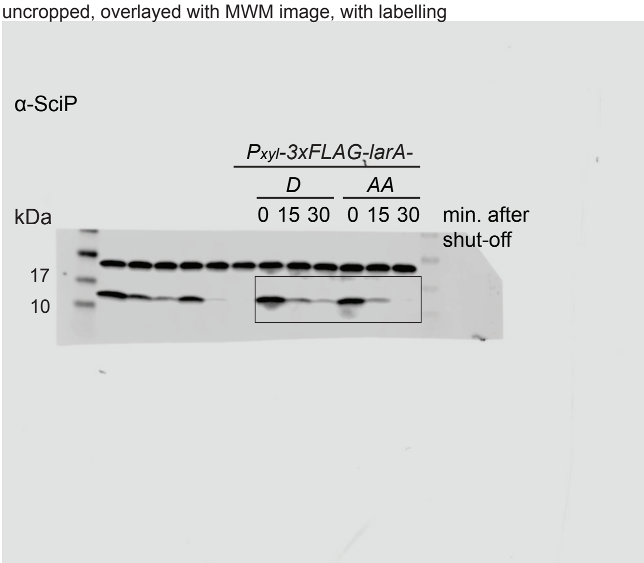

Figure 4d
anti-FLAG,
Input

uncropped

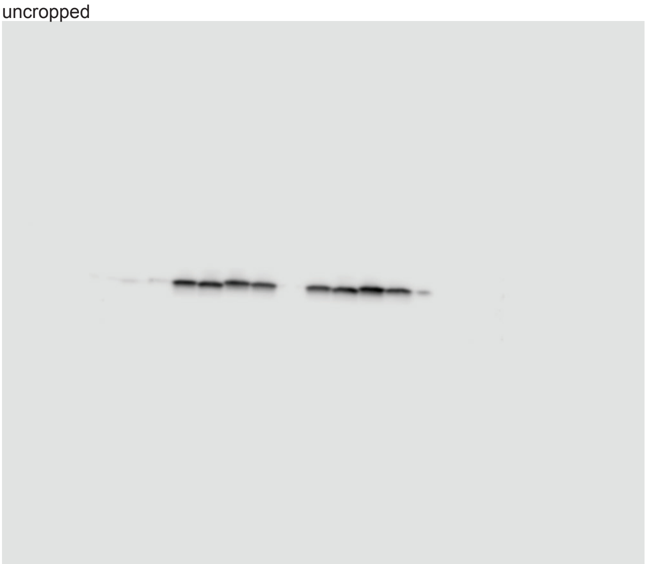

uncropped, with labelling

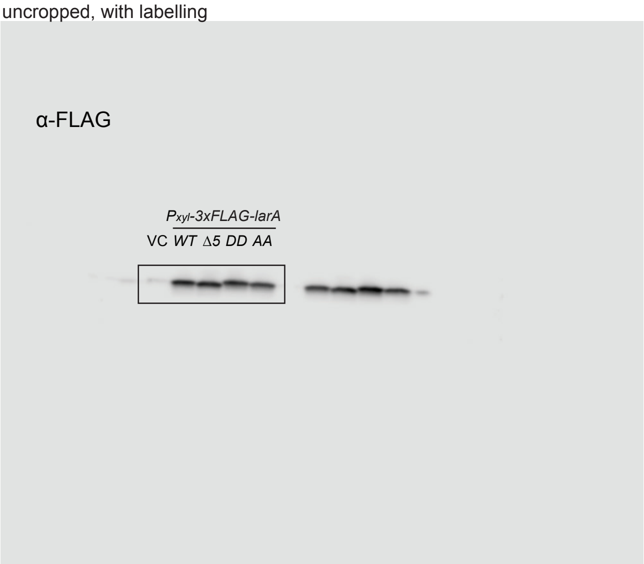

Figure 4d
anti-FLAG IP

uncropped

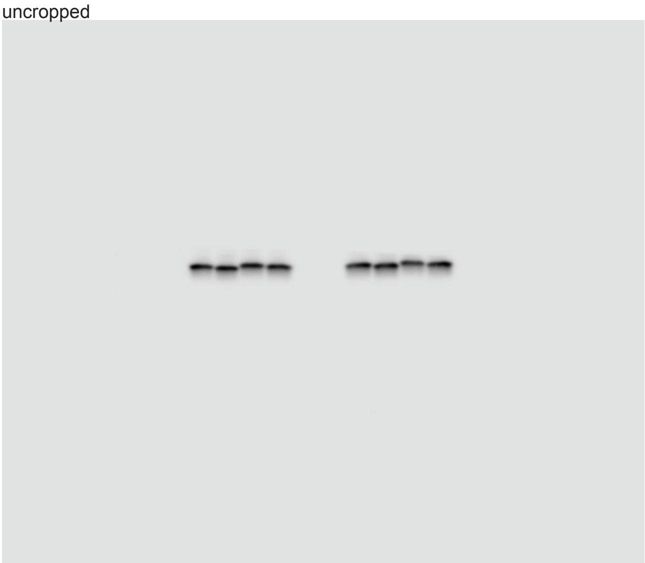

uncropped, overlayed with MWM image, with labelling

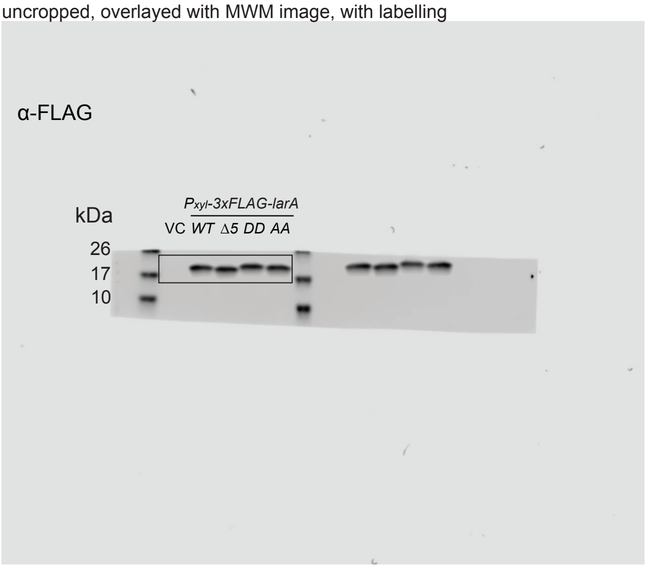

Figure 4d
anti-lon,
Input

uncropped

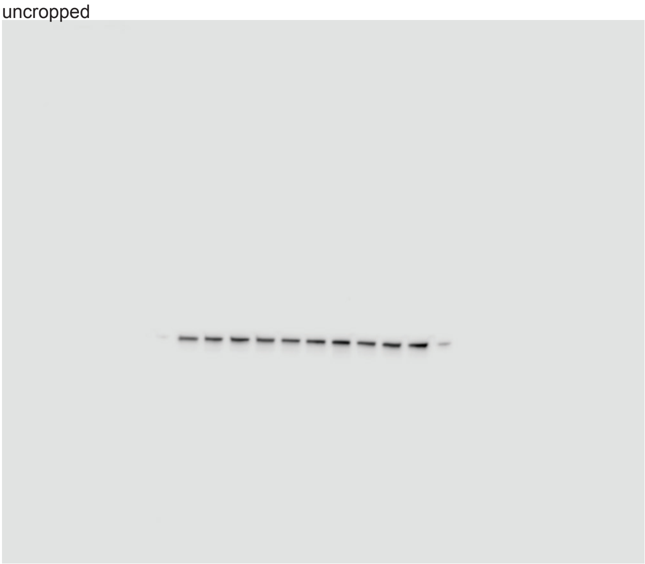

uncropped, overlayed with MWM image

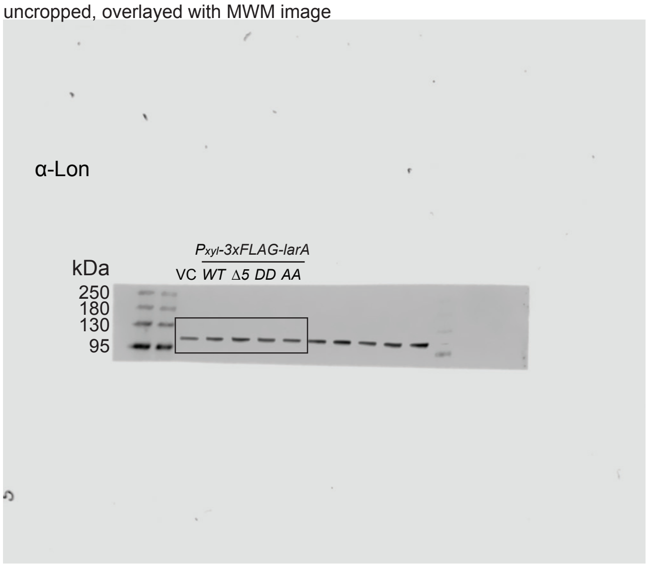

Figure 4d
anti-lon,
anti-FLAG IP

uncropped

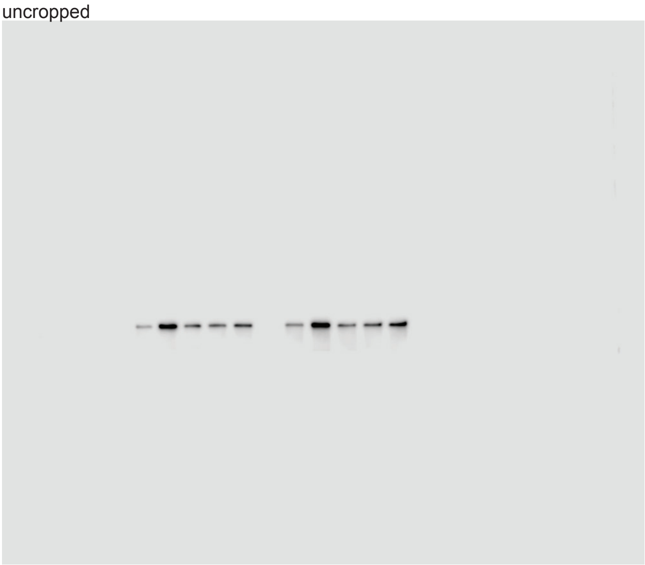

uncropped, overlayed with MWM image

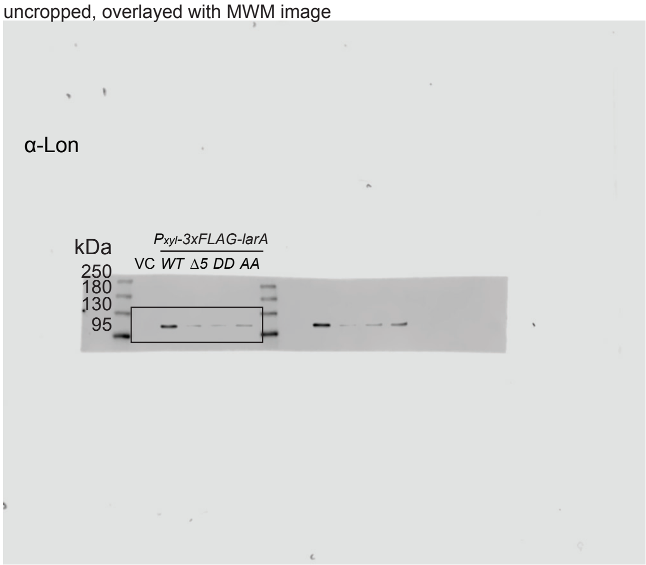

Figure 4f
His-SciP
−LarA

uncropped

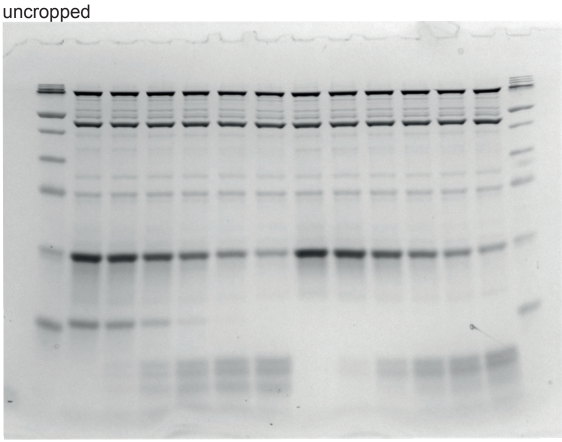

uncropped with cropmarks, labelled bands

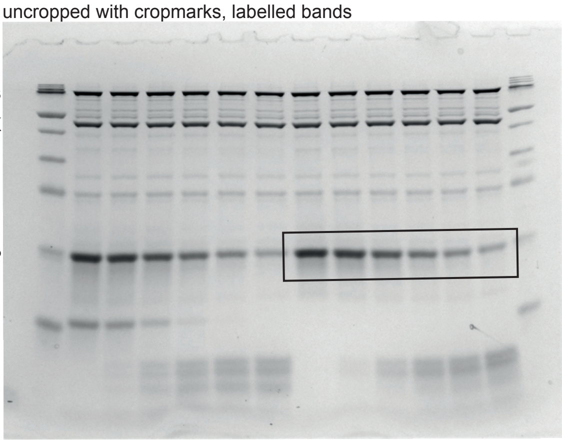

Figure 4f
His-SciP
+LarA^{WT}

uncropped

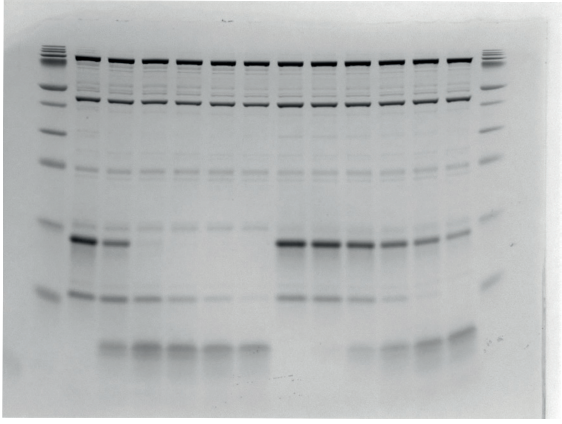

uncropped with cropmarks, labelled bands

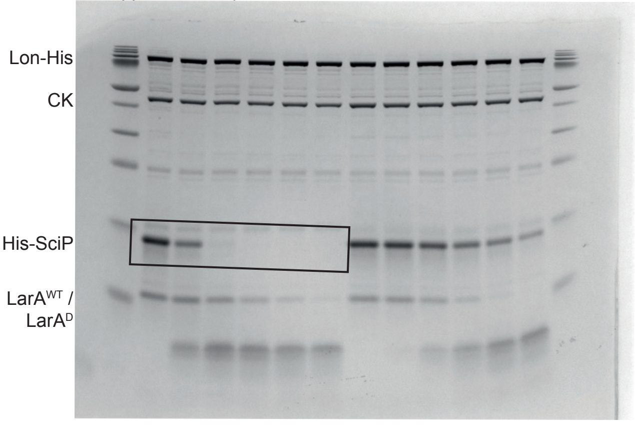

Figure 4f
His-SciP
+LarA^{AA}

uncropped

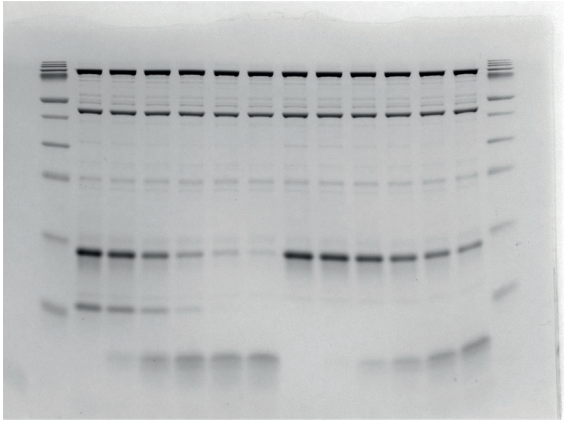

uncropped with cropmarks, labelled bands

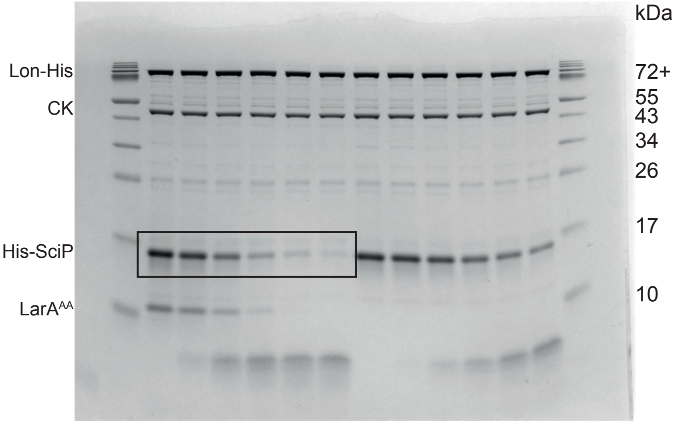

Figure 4f
His-SciP
+LarA^{D}

uncropped

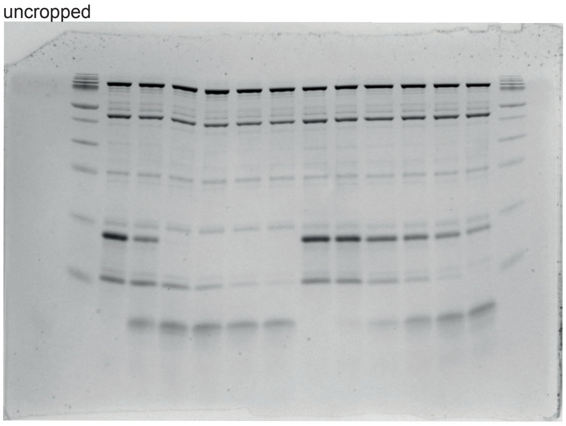

uncropped with cropmarks, labelled bands

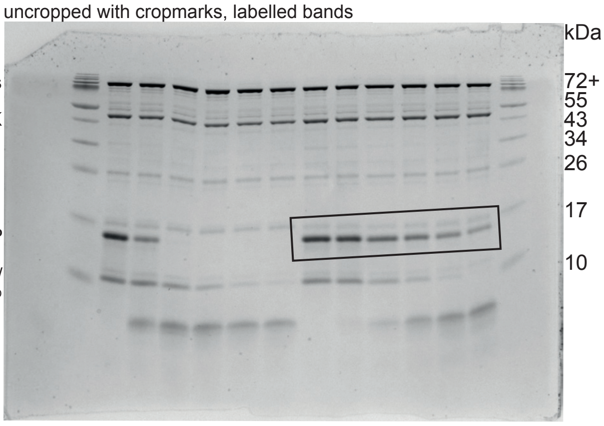

Figure 4f
His-SciP
+LarA^{DD}

uncropped

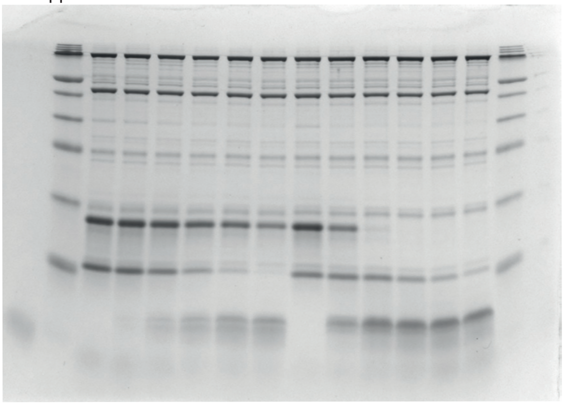

uncropped with cropmarks, labelled bands

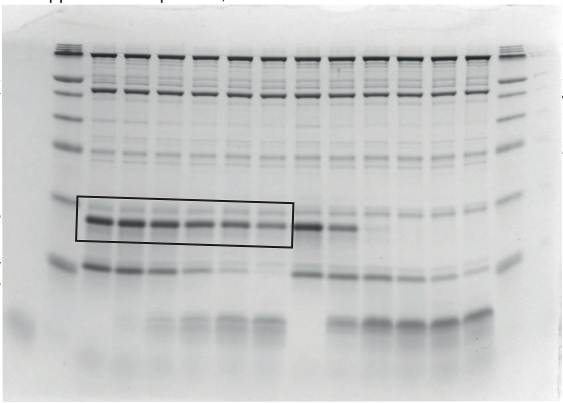

Figure 4f
His-SciP
+LarA$^{\Delta 5}$

uncropped

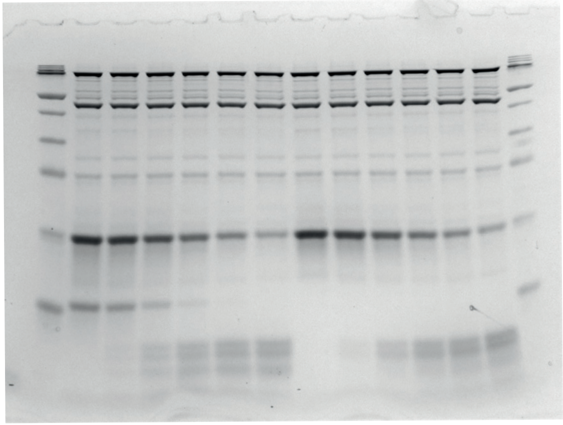

uncropped with cropmarks, labelled bands

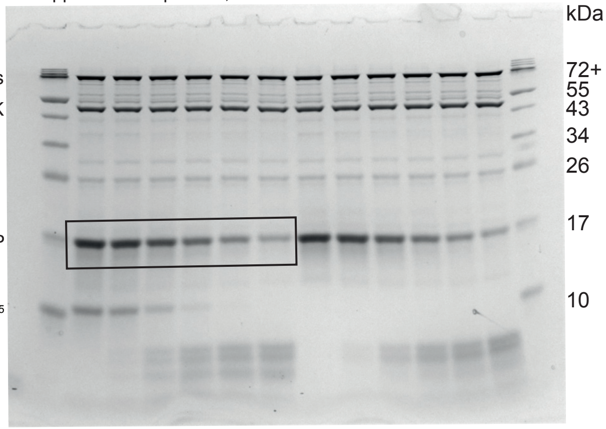

Figure 4g
LarA^{WT}

uncropped

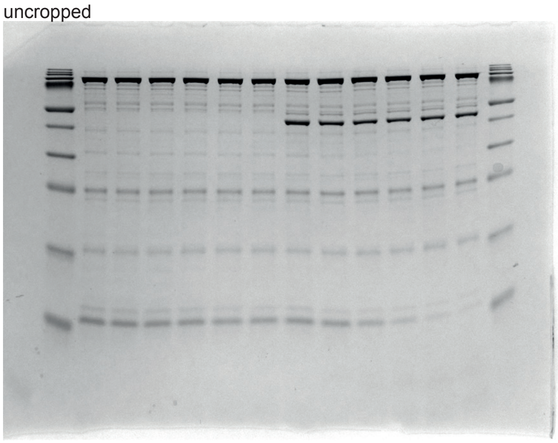

uncropped with cropmarks, labelled bands

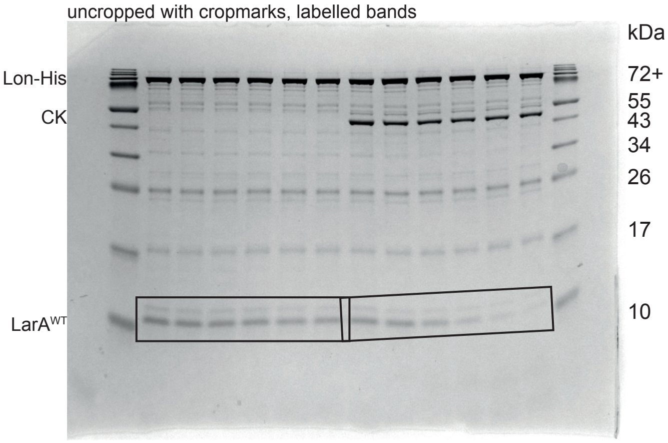

Figure 4g
LarA^{AA}

uncropped

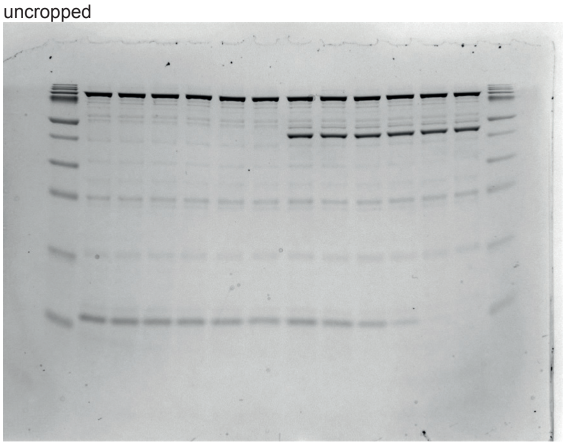

uncropped with cropmarks, labelled bands

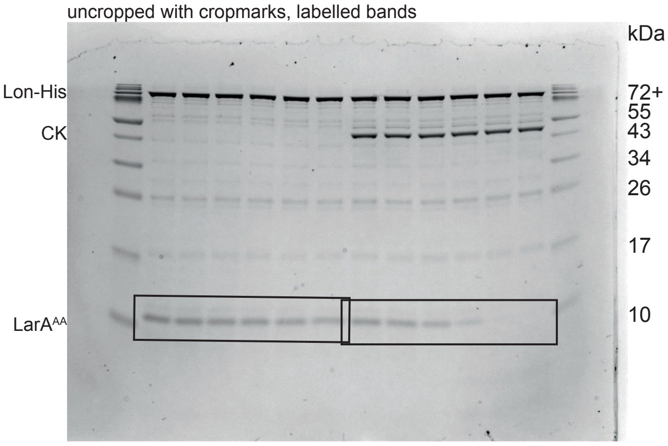

Figure 4g
LarA^{D}

uncropped

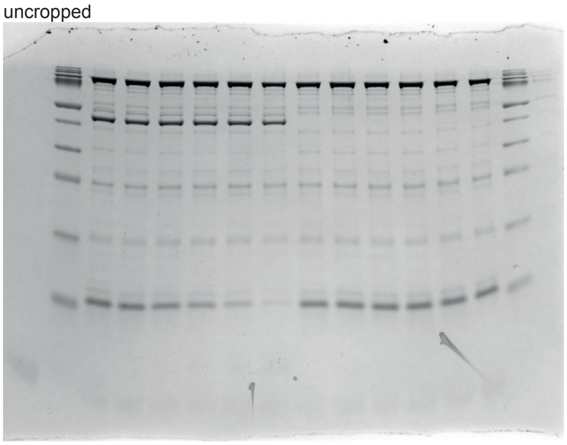

uncropped with cropmarks, labelled bands

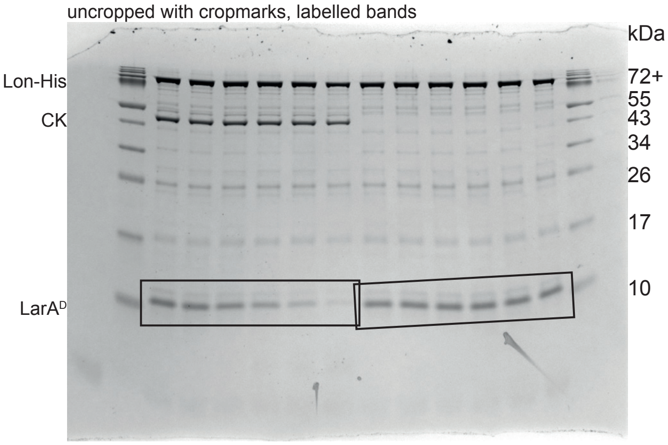

Figure 4g
LarA^{DD}

uncropped

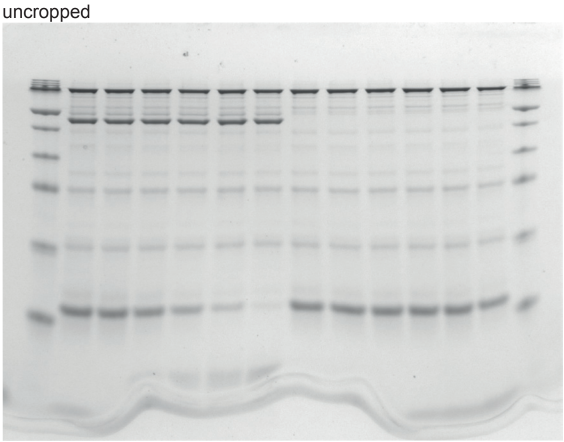

uncropped with cropmarks, labelled bands

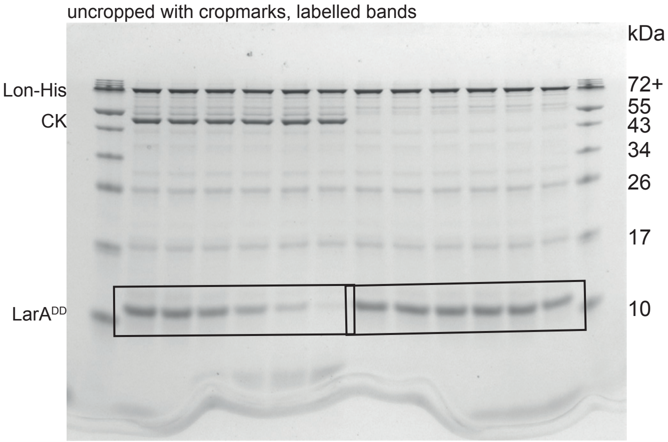

Figure 4g
LarA$^{\Delta 5}$

uncropped

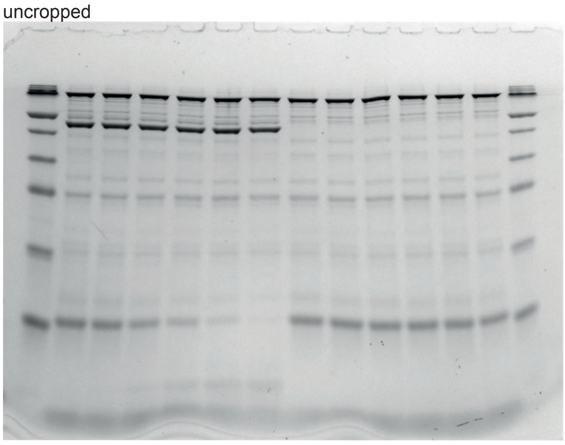

uncropped with cropmarks, labelled bands

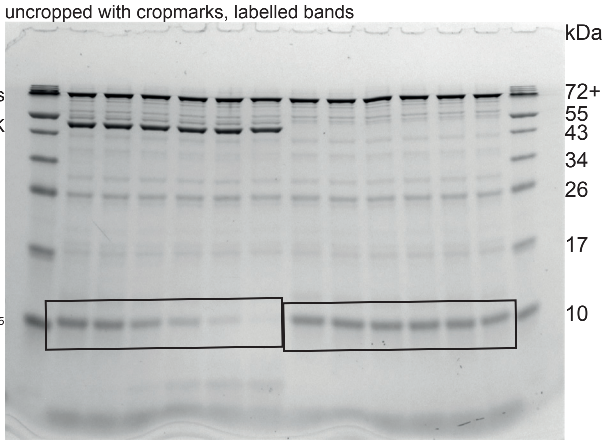

Supplement: Supplementary file 6 — Source Data [file 41467_2023_43385_MOESM6_ESM.zip › Figure 4 - Uncropped blots and gels.pdf]
