# Supplementary material for: The heat shock protein LarA activates the Lon protease in response to proteotoxic stress
Source: Nat Commun. 2023 Nov 22;14:7636. doi: 10.1038/s41467-023-43385-x (PMC10665427; doi:10.1038/s41467-023-43385-x)

Uncroppped Western Blot and Gel images with molecular weight markers

Figure 6a
His-SciP

uncropped

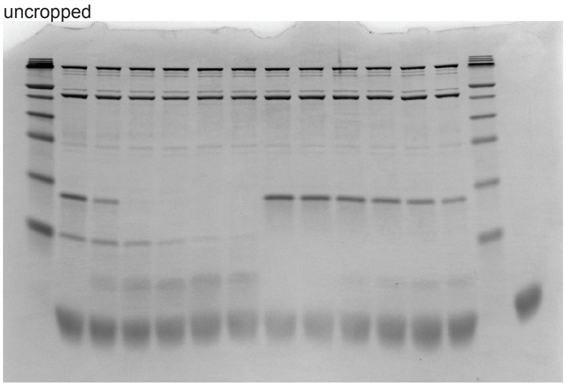

uncropped with cropmarks, labelled bands

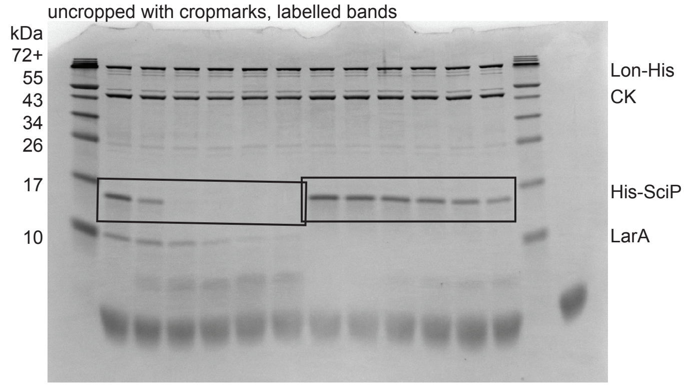

Figure 6a
CcrM

uncropped

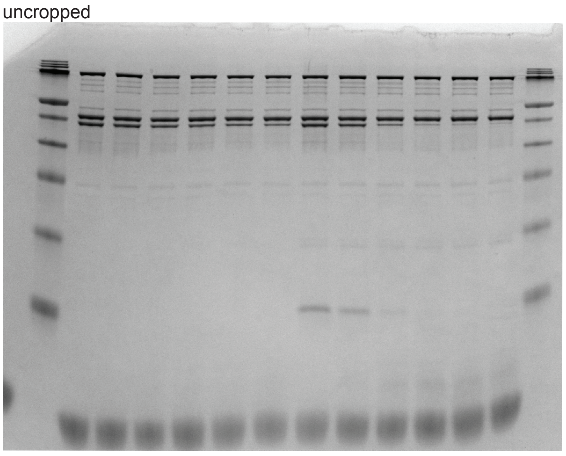

uncropped with cropmarks, labelled bands

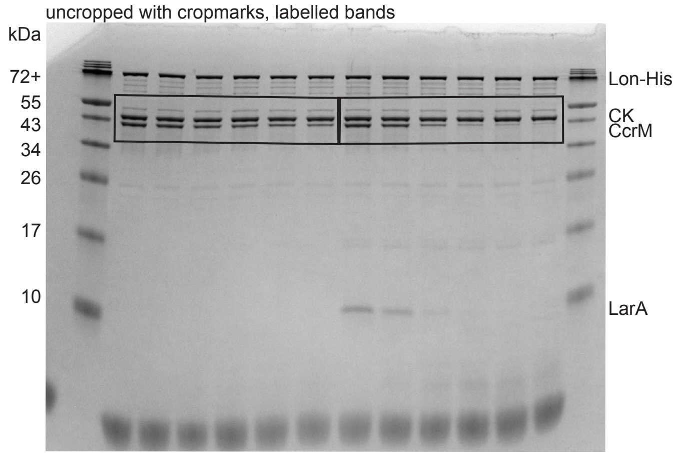

Figure 6a
FliX

uncropped

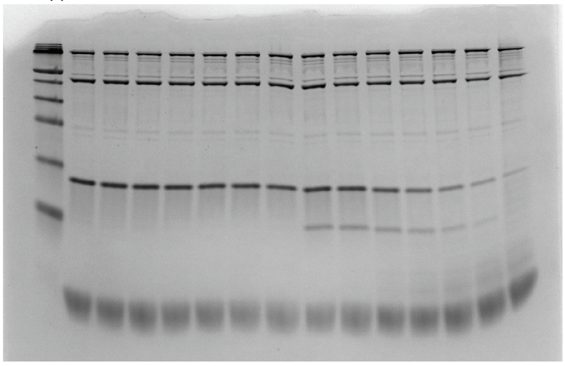

uncropped with cropmarks, labelled bands

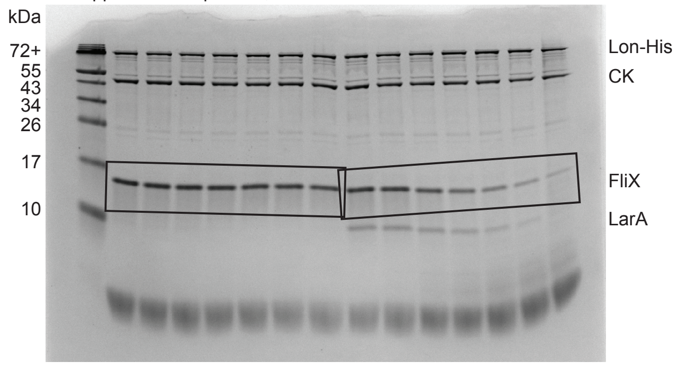

Figure 6a
FliK-C

uncropped

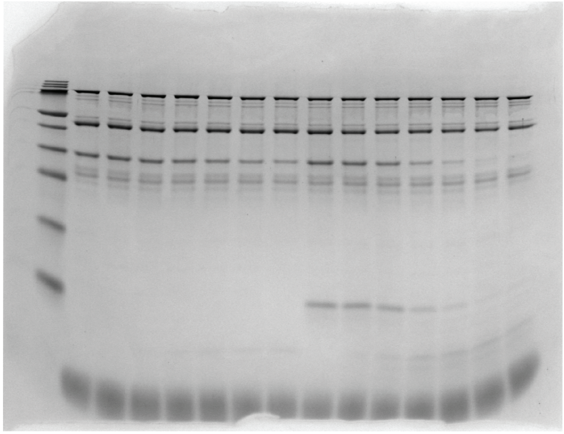

uncropped with cropmarks, labelled bands

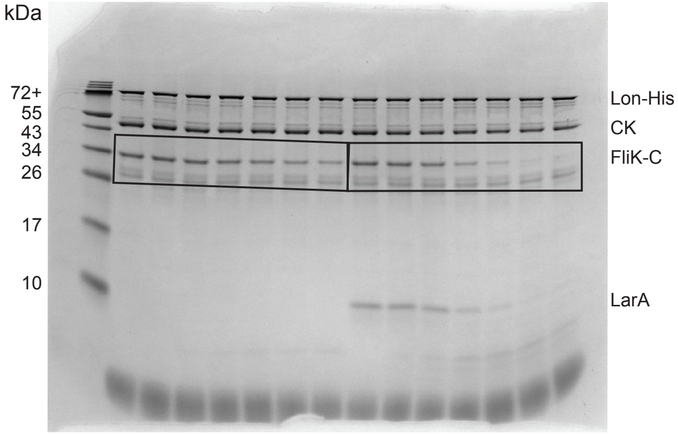

Figure 6a
DnaA

uncropped

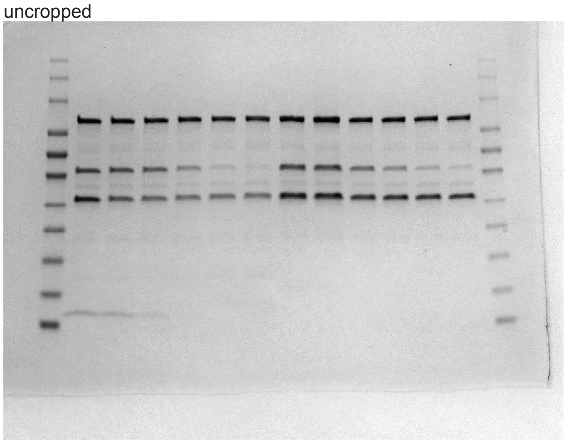

uncropped with cropmarks, labelled bands

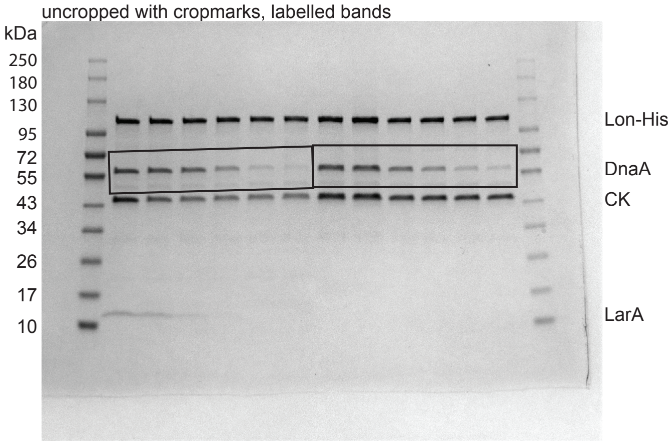

Figure 6a
β-casein

uncropped

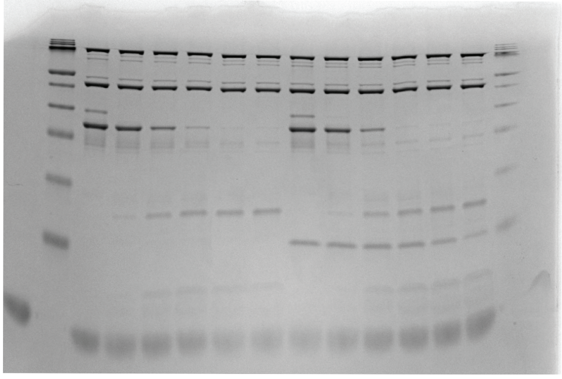

uncropped with cropmarks, labelled bands

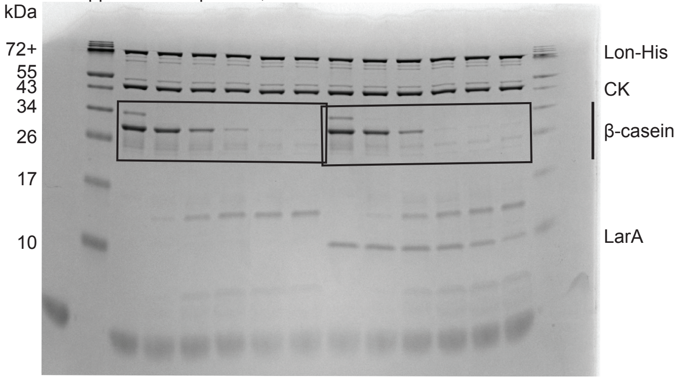

Figure 6a
6xHis-titin-β20^{CM}

uncropped

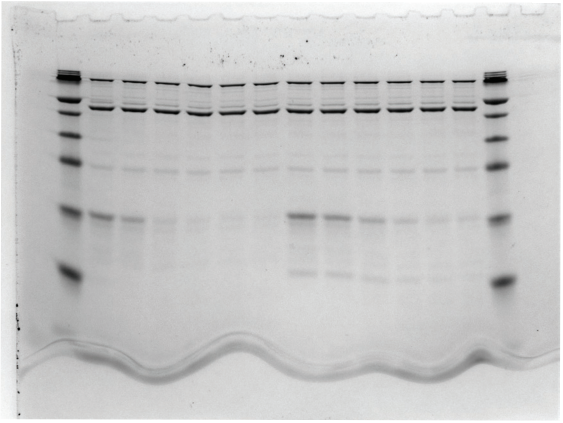

uncropped with cropmarks, labelled bands

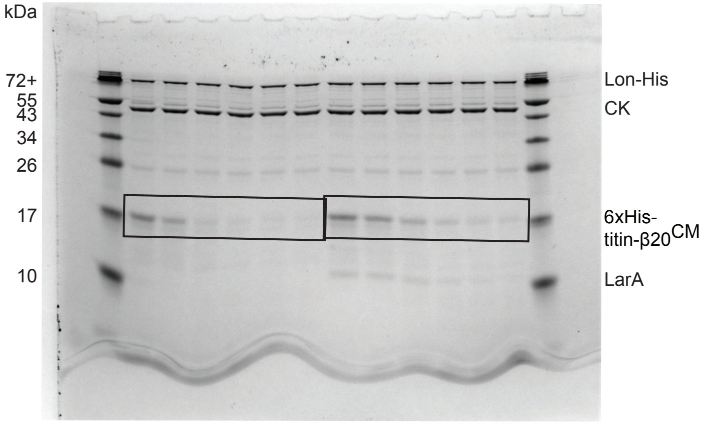

Figure 6d
anti-FliK-C

uncropped

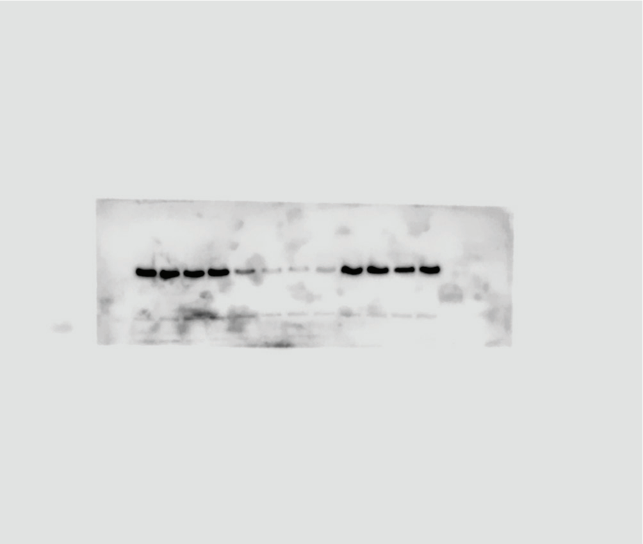

uncropped, overlayed with MWM image, with labelling

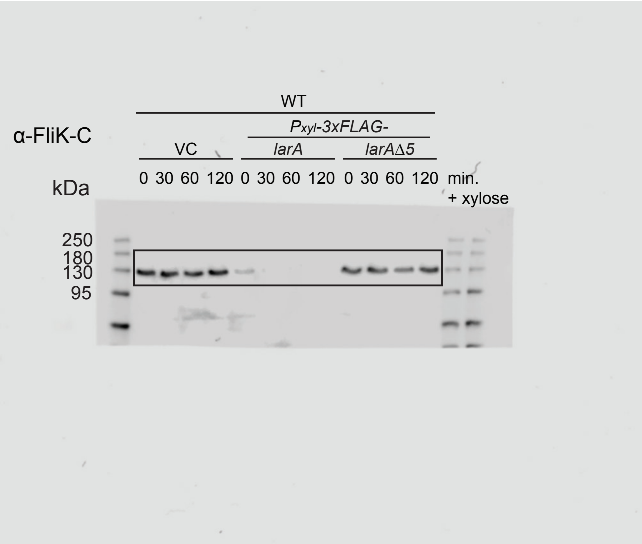

Figure 6d
anti-FLAG

uncropped

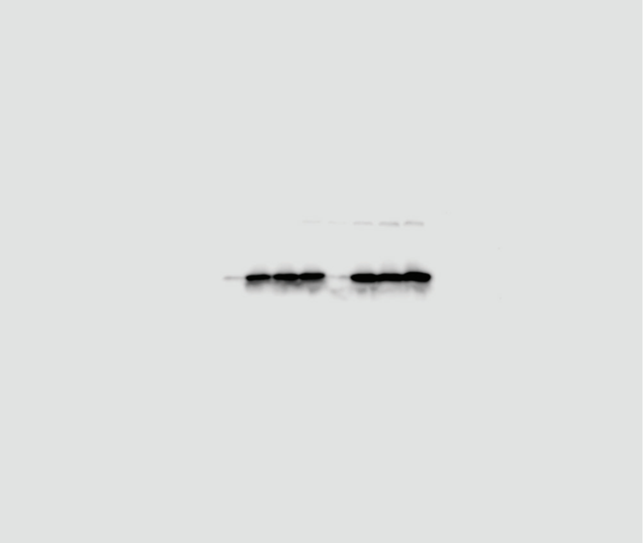

uncropped, overlayed with MWM image, with labelling

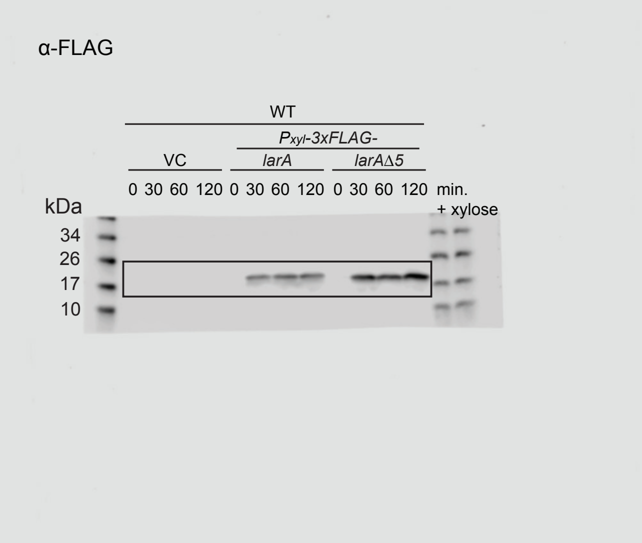

Supplement: Supplementary file 6 — Source Data [file 41467_2023_43385_MOESM6_ESM.zip › Figure 6 - Uncropped blots and gels.pdf]
