# Supplementary material for: The heat shock protein LarA activates the Lon protease in response to proteotoxic stress
Source: Nat Commun. 2023 Nov 22;14:7636. doi: 10.1038/s41467-023-43385-x (PMC10665427; doi:10.1038/s41467-023-43385-x)

Uncroppped Western Blot and Gel images with molecular weight markers

Figure 7c
anti-DnaK

uncropped

uncropped, overlayed with MWM image, with labelling

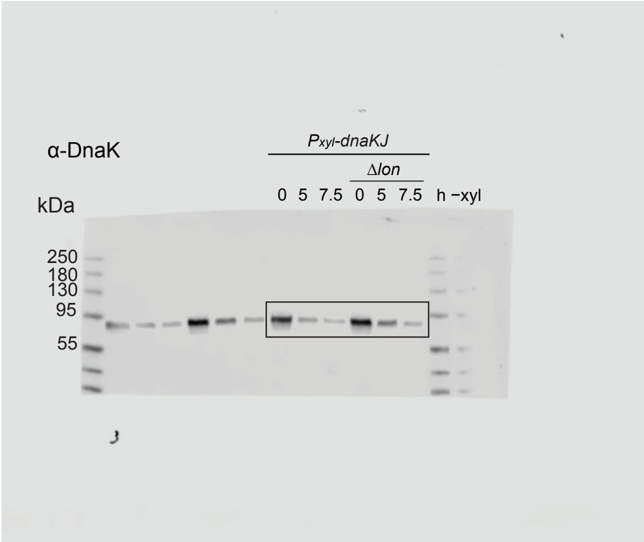

Figure 7c
anti-LarA

uncropped

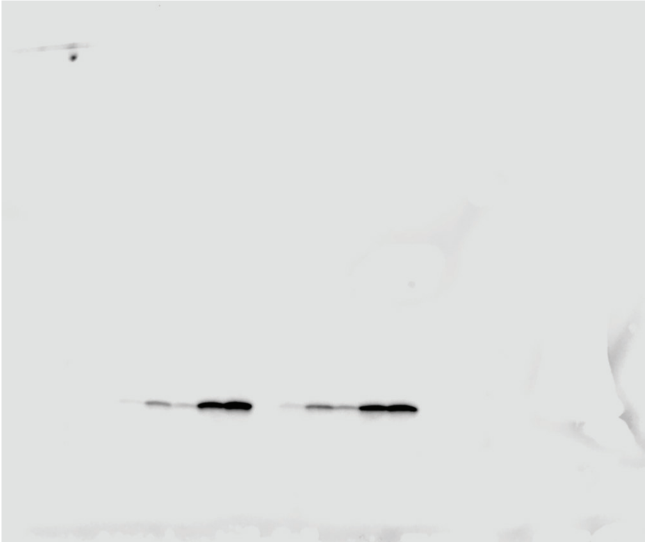

uncropped, overlayed with MWM image, with labelling

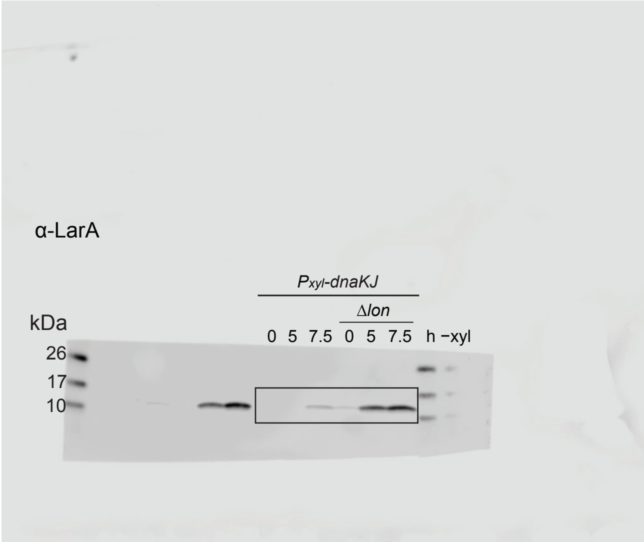

Figure 7d
anti-LarA,
upper panel

uncropped

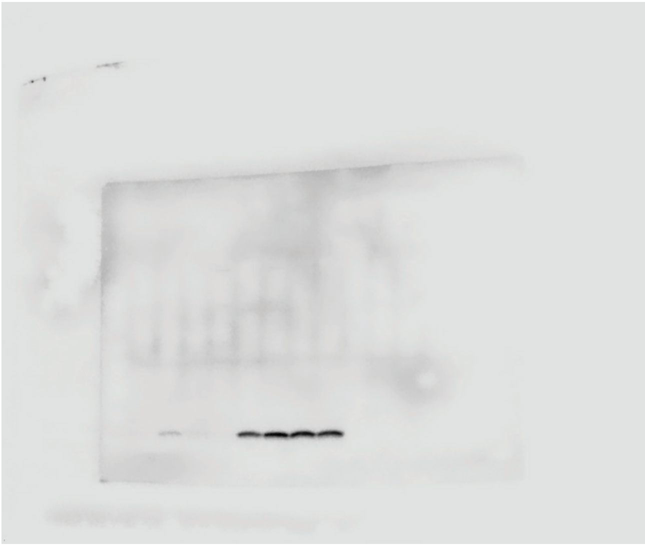

uncropped, overlayed with MWM image, with labelling

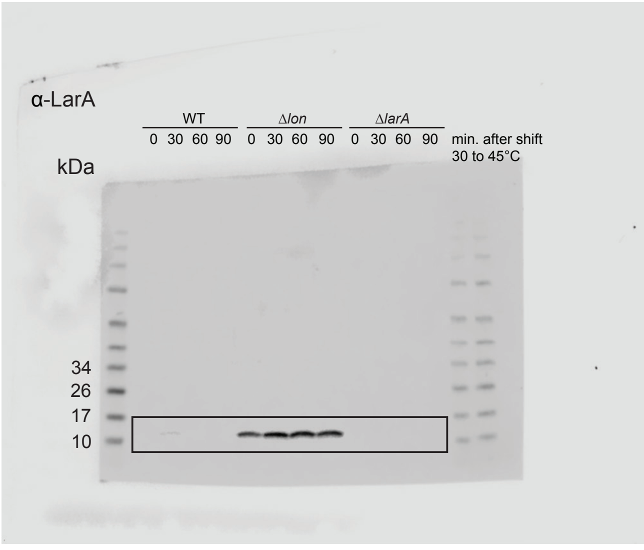

Figure 7d
anti-LarA,
lower panel

uncropped

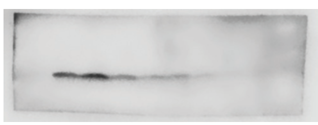

uncropped, overlayed with MWM image, with labelling

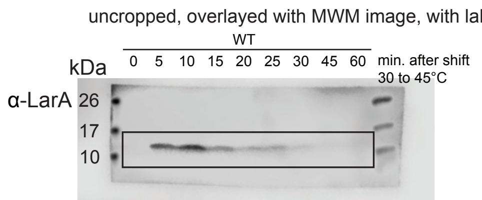

Figure 7e
anti-LarA

uncropped

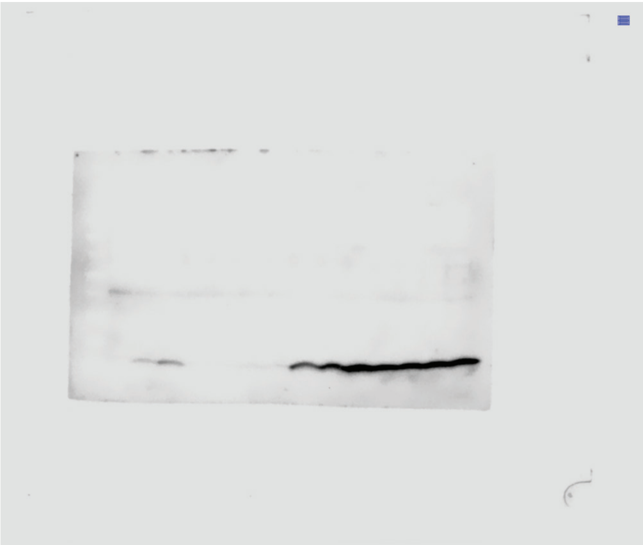

uncropped, overlayed with MWM image, with labelling

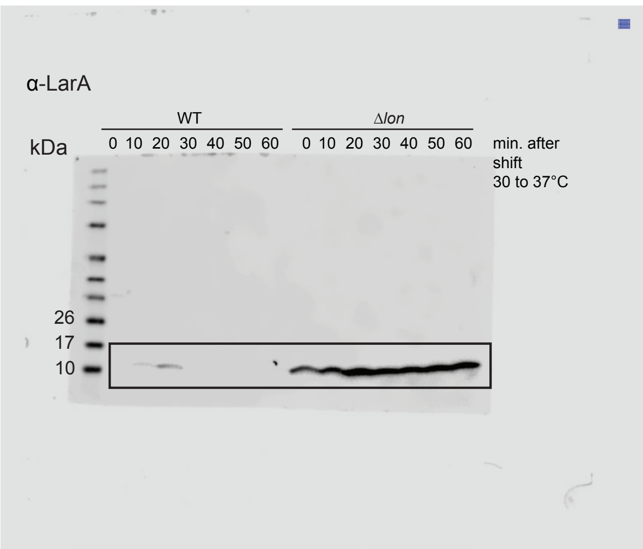

Figure 7f
anti-LarA

uncropped

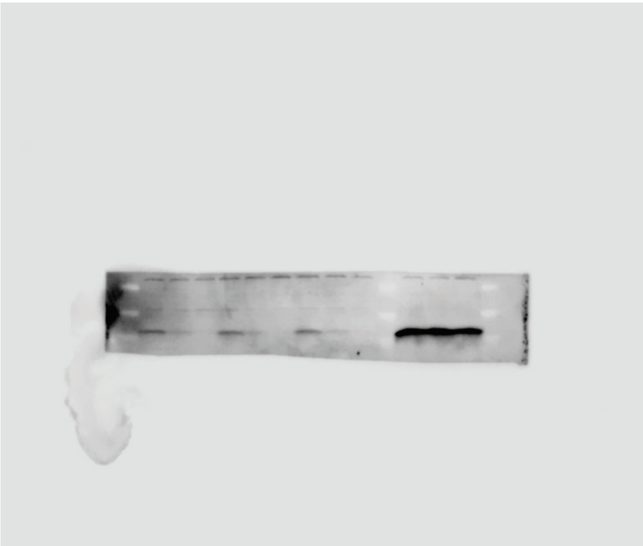

uncropped, overlayed with MWM image, with labelling

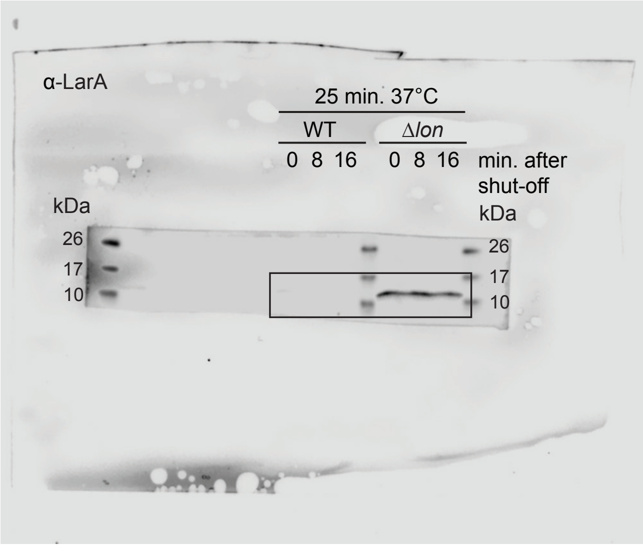

Figure 7g
anti-LarA

uncropped

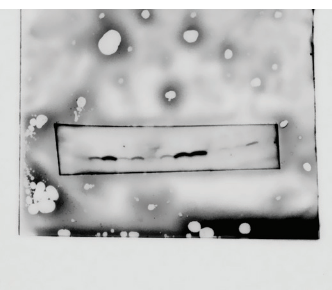

uncropped, overlayed with MWM image

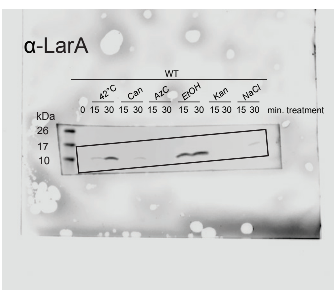

Supplement: Supplementary file 6 — Source Data [file 41467_2023_43385_MOESM6_ESM.zip › Figure 7 - Uncropped blots and gels.pdf]
