# Supplementary material for: The heat shock protein LarA activates the Lon protease in response to proteotoxic stress
Source: Nat Commun. 2023 Nov 22;14:7636. doi: 10.1038/s41467-023-43385-x (PMC10665427; doi:10.1038/s41467-023-43385-x)

Uncroppped Western Blot and Gel images with molecular weight markers

Supplementary Figure 3

Supplementary Figure 3a, upper panel

uncropped

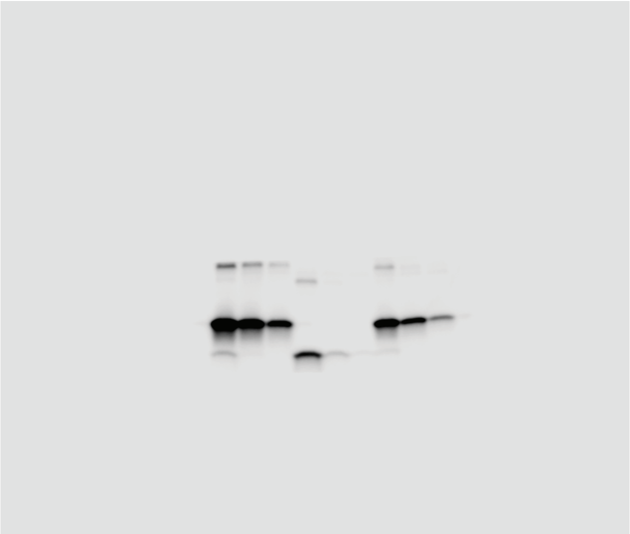

uncropped, overlayed with MWM image, and labelling

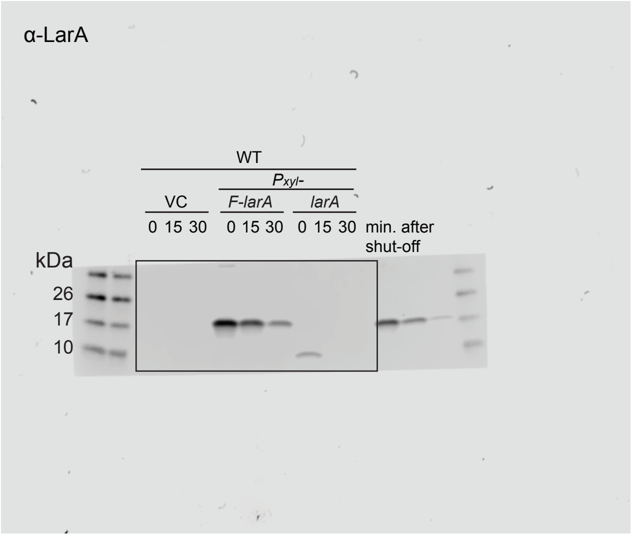

Supplementary Figure 3a, lower panel

uncropped

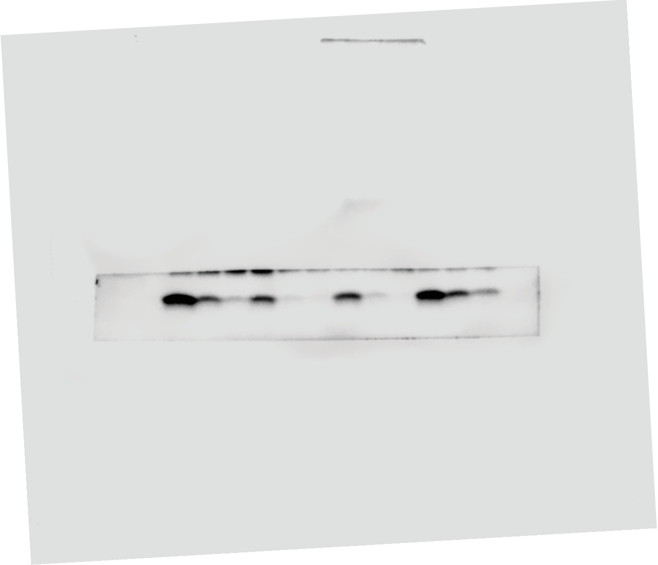

uncropped, overlayed with MWM image, and labelling

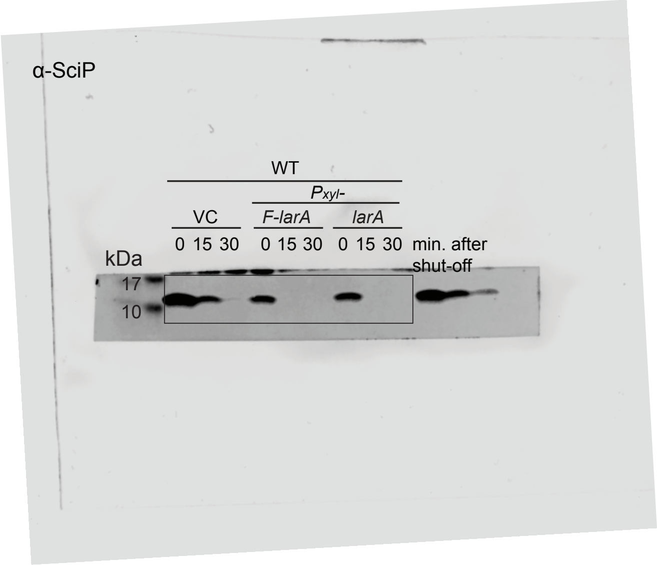

Uncroppped Western Blot and Gel images with molecular weight markers

Supplementary Figure 3

Supplementary Figure 3b

uncropped

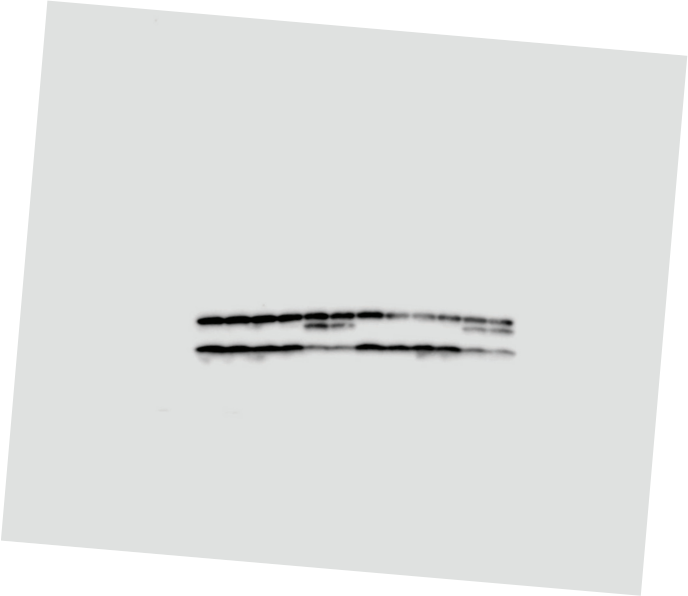

uncropped, overlayed with MWM image, and labelling

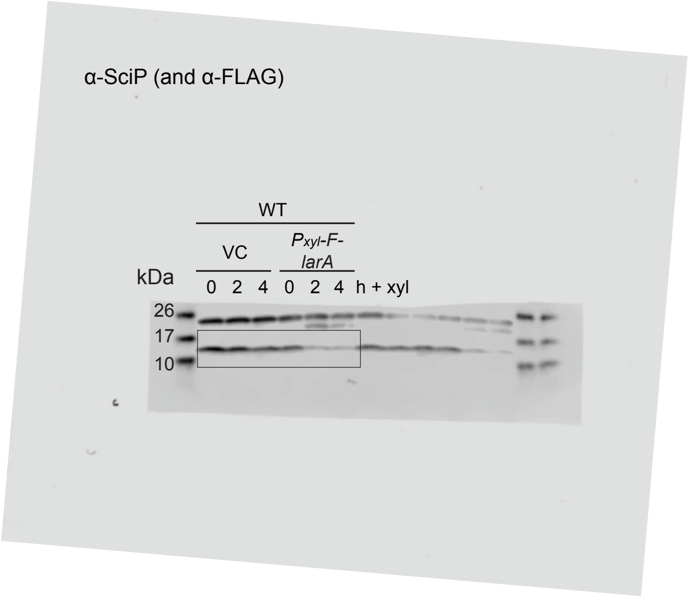

Supplement: Supplementary file 6 — Source Data [file 41467_2023_43385_MOESM6_ESM.zip › Supplementary Figure 3 - Uncropped blots and gels.pdf]
