# Supplementary material for: The heat shock protein LarA activates the Lon protease in response to proteotoxic stress
Source: Nat Commun. 2023 Nov 22;14:7636. doi: 10.1038/s41467-023-43385-x (PMC10665427; doi:10.1038/s41467-023-43385-x)

Uncroppped Western Blot and Gel images with molecular weight markers

Supplementary Figure 7

Supplementary Figure 7a

uncropped

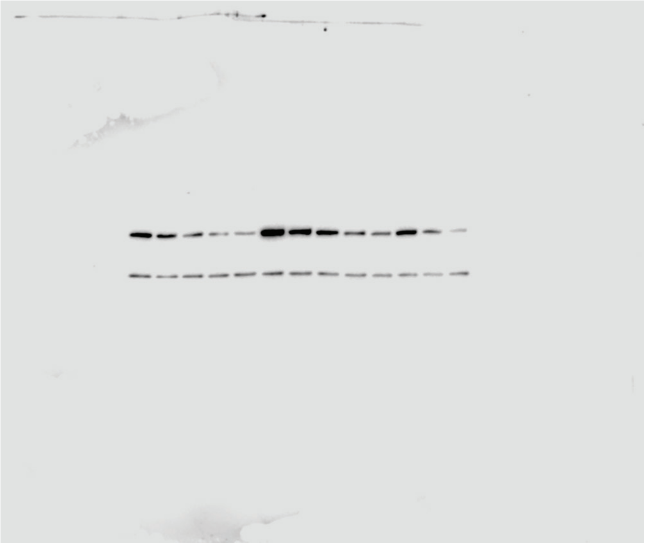

uncropped, overlayed with MWM image, and labelling

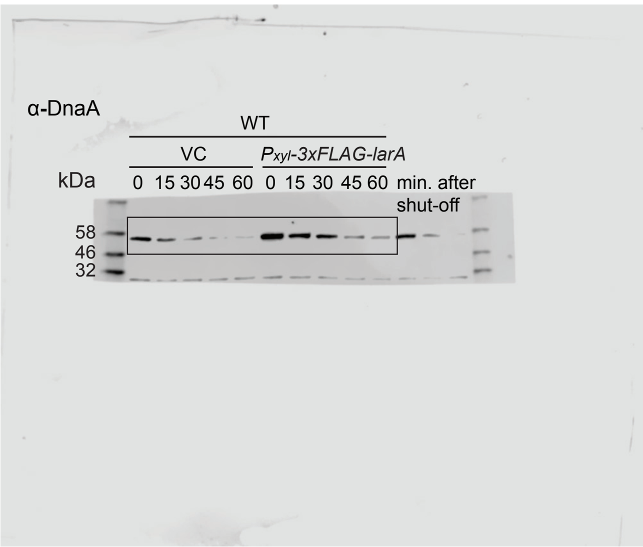

Uncropppped Western Blot and Gel images with molecular weight markers

Supplementary Figure 7

Supplementary Figure 7c, upper panel

uncropped

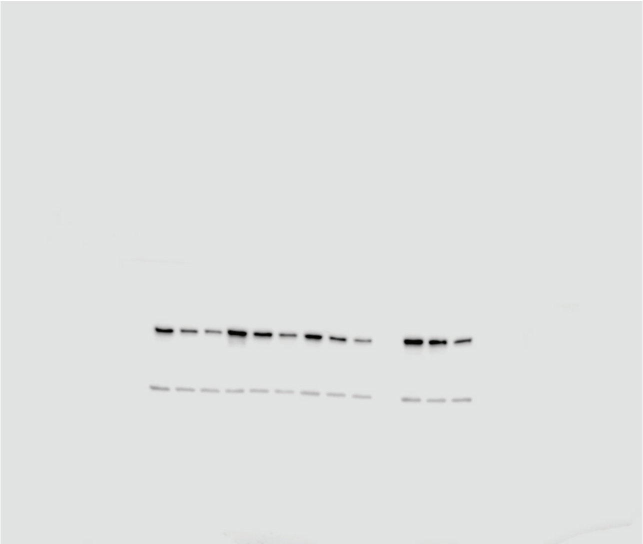

uncropped, overlayed with MWM image, and labelling

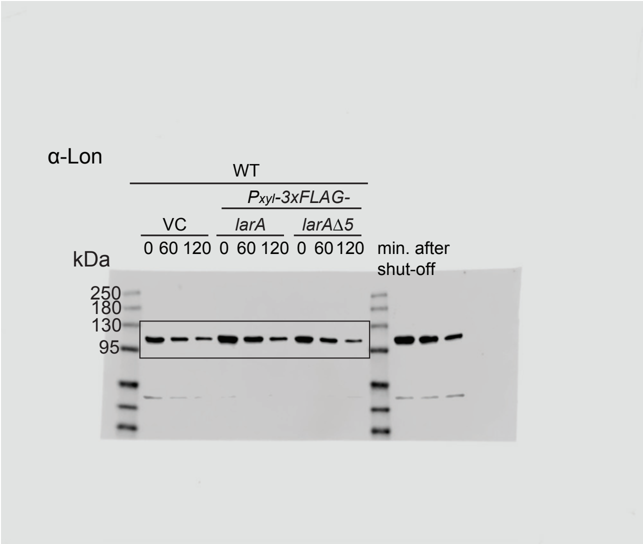

Supplementary Figure 7c, lower panel

uncropped

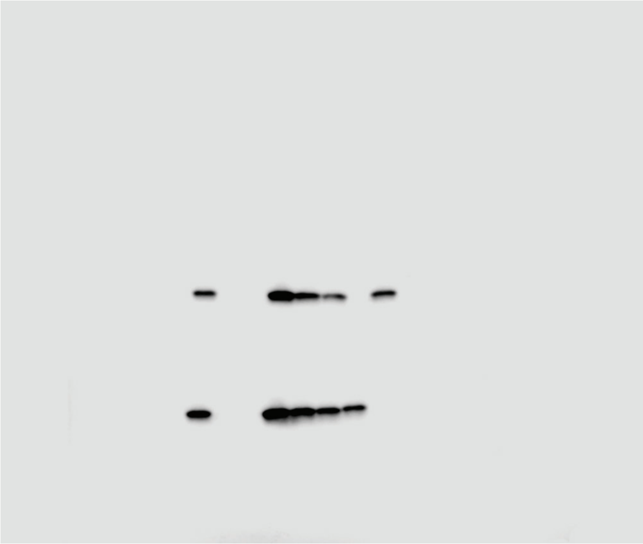

uncropped, overlayed with MWM image, and labelling

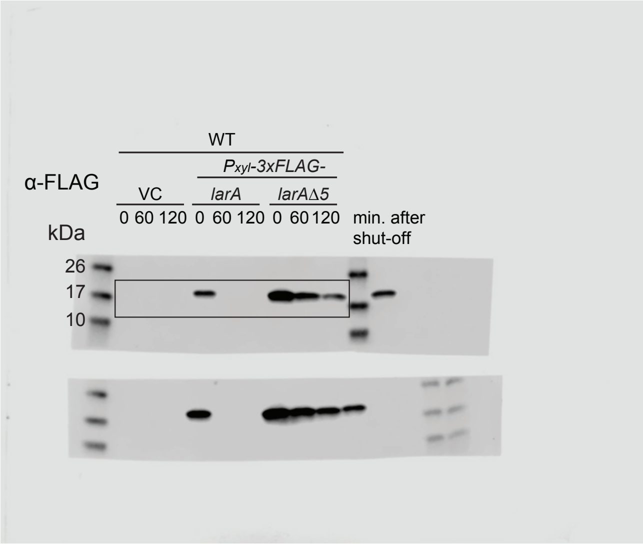

Supplement: Supplementary file 6 — Source Data [file 41467_2023_43385_MOESM6_ESM.zip › Supplementary Figure 7 - Uncropped blots and gels.pdf]
