# Supplementary figures and images for: The heat shock protein LarA activates the Lon protease in response to proteotoxic stress
Source: Nat Commun. 2023 Nov 22;14:7636. doi: 10.1038/s41467-023-43385-x (PMC10665427; doi:10.1038/s41467-023-43385-x)

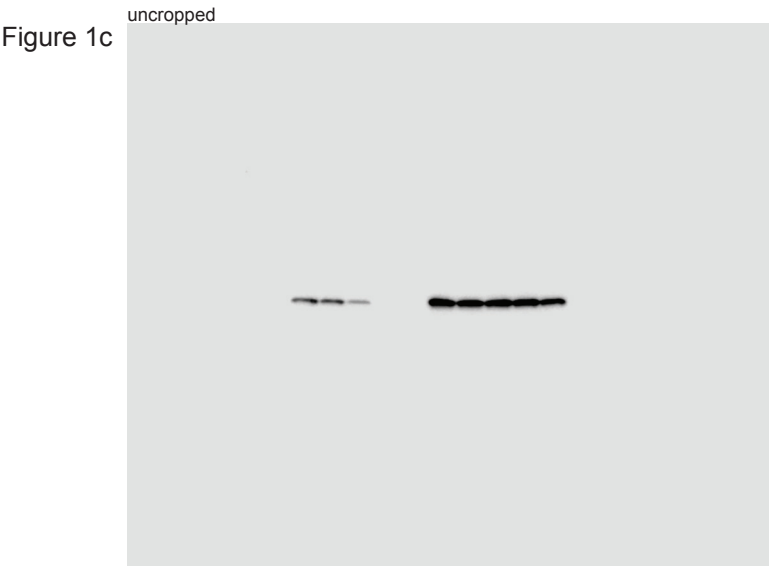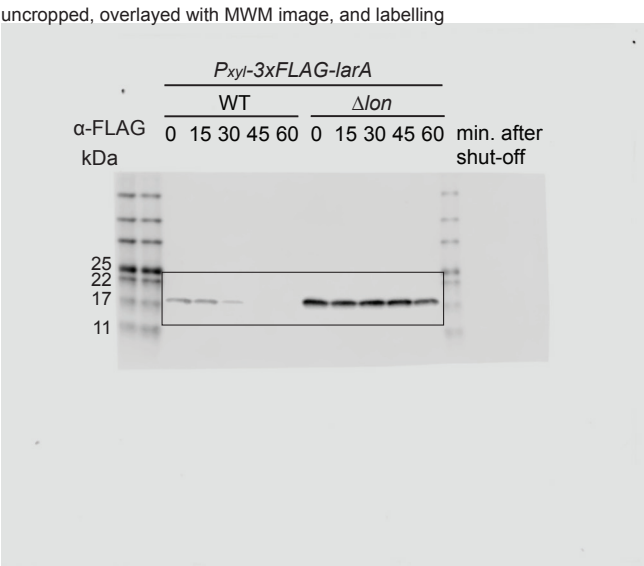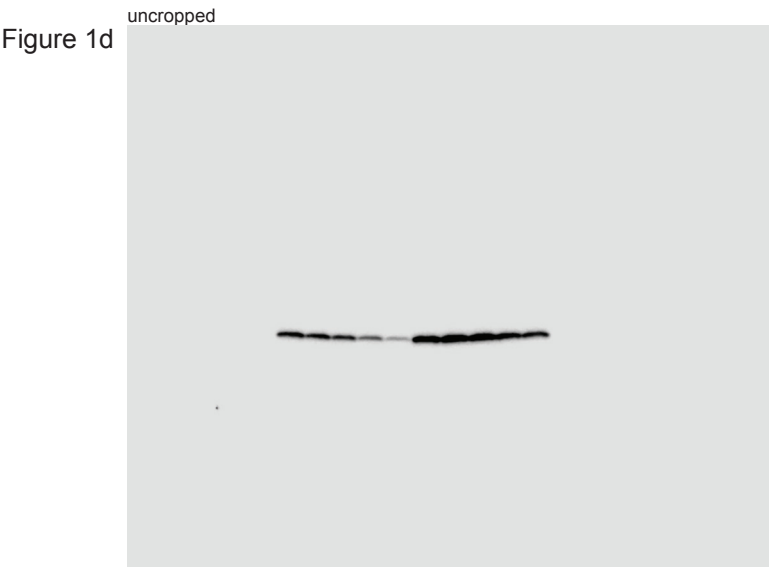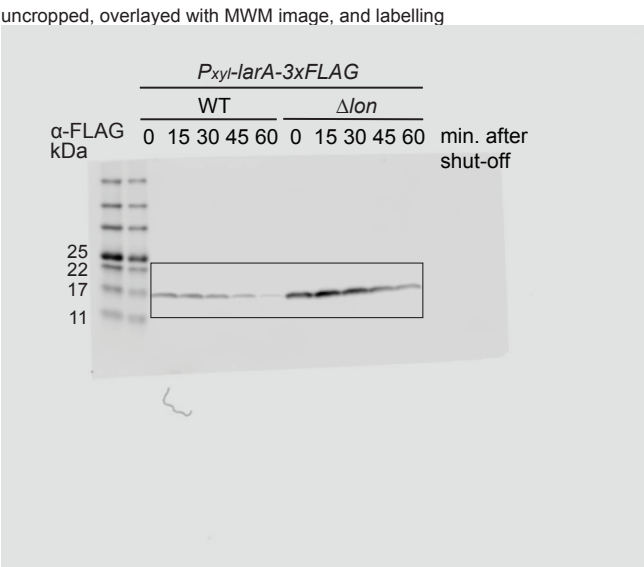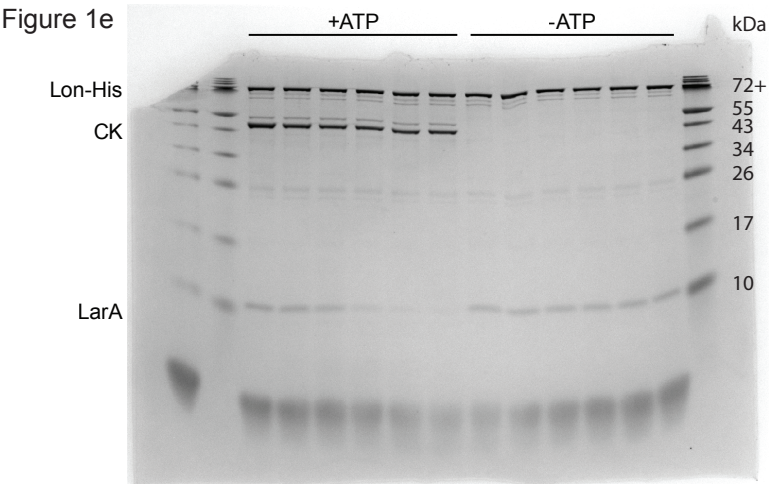

Supplement: Supplementary file 6 — Source Data [file 41467_2023_43385_MOESM6_ESM.zip › Figure 1 - Uncropped blots and gels.pdf]

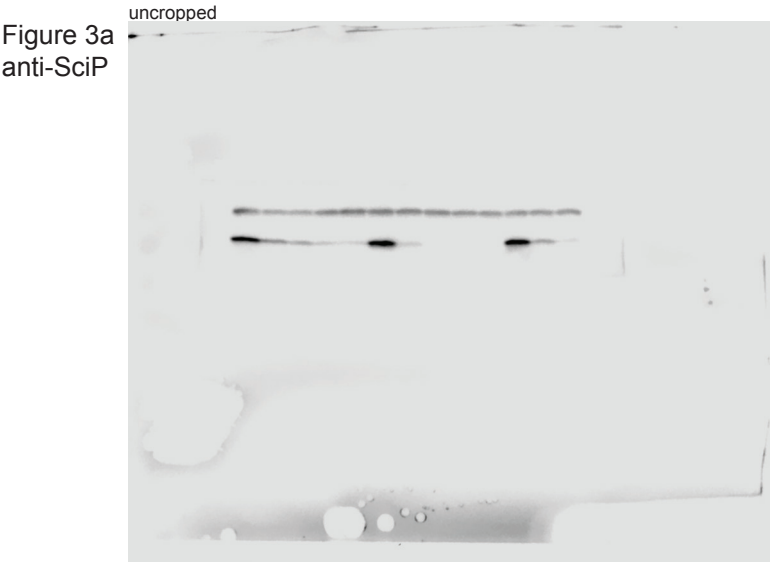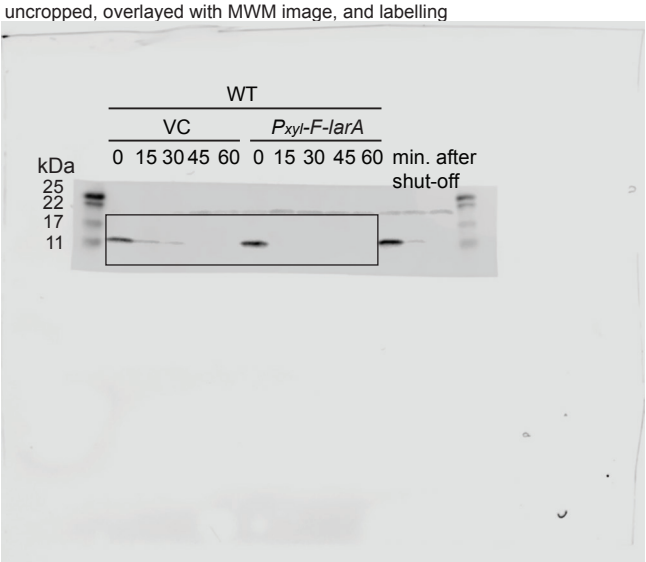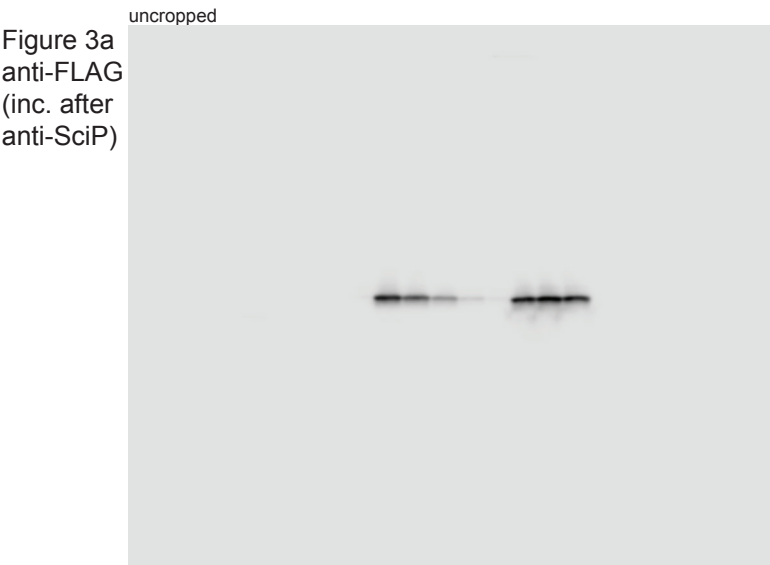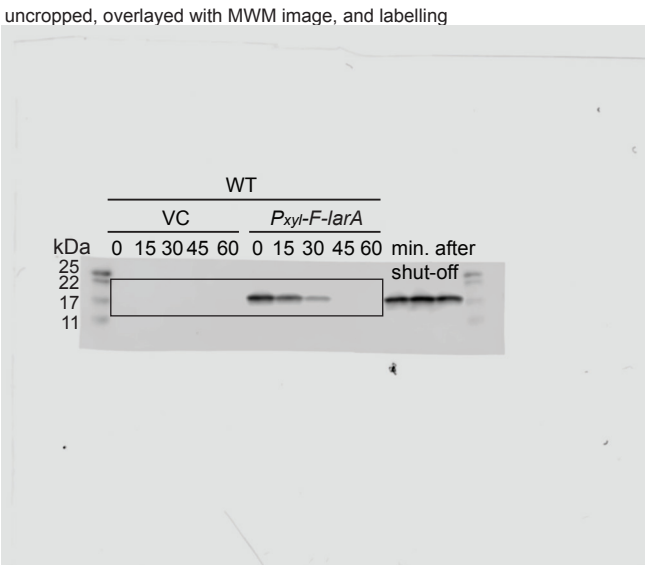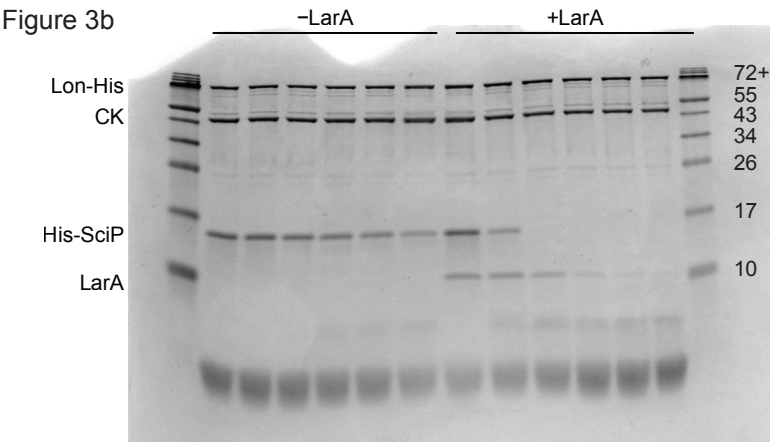

Supplement: Supplementary file 6 — Source Data [file 41467_2023_43385_MOESM6_ESM.zip › Figure 3 - Uncropped blots and gels.pdf]

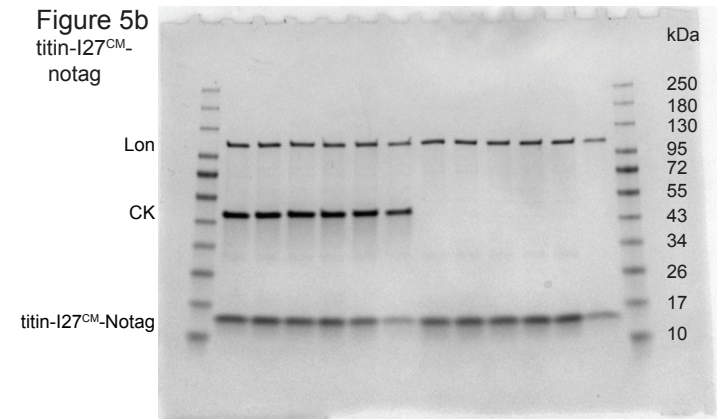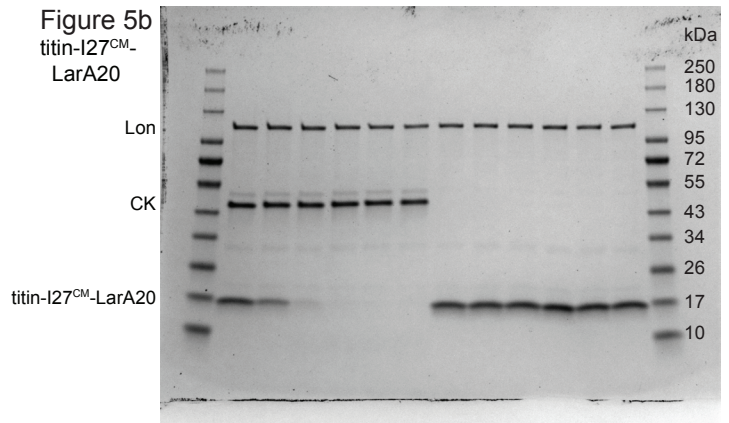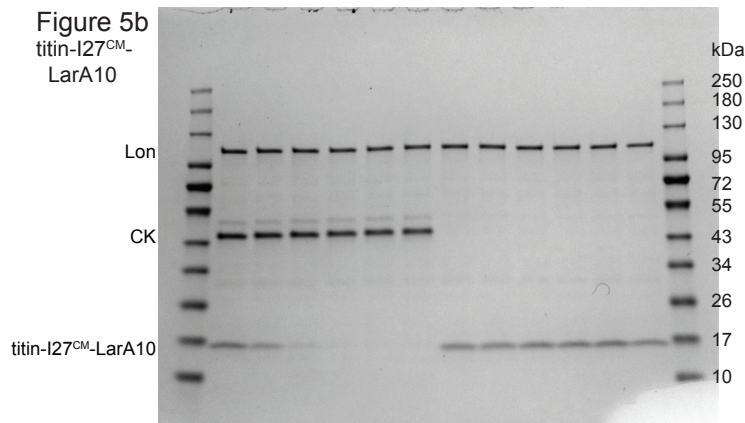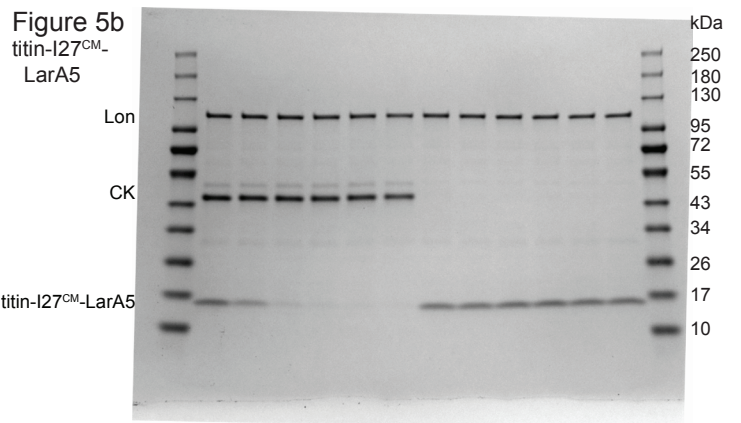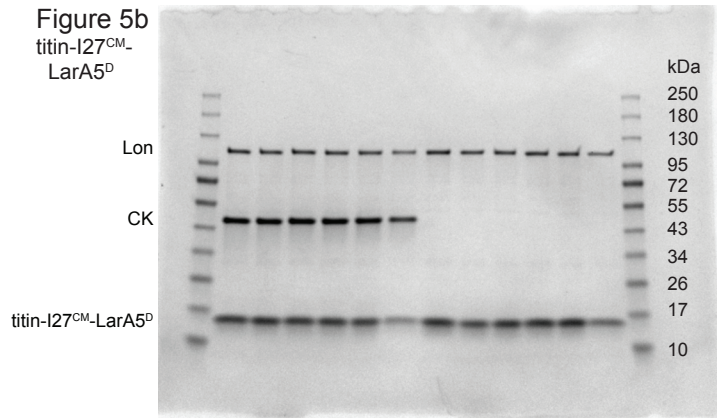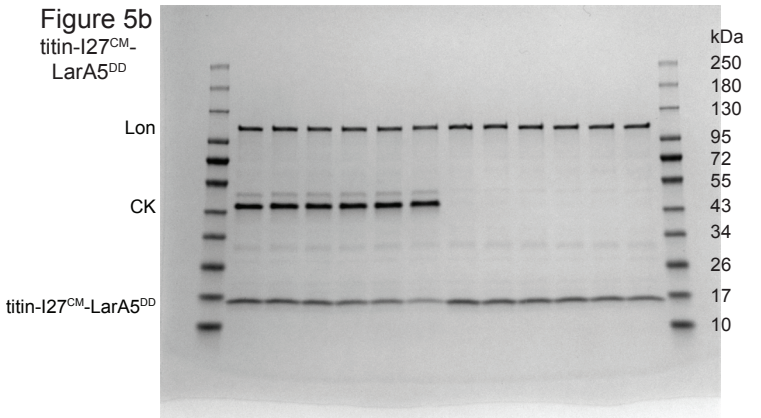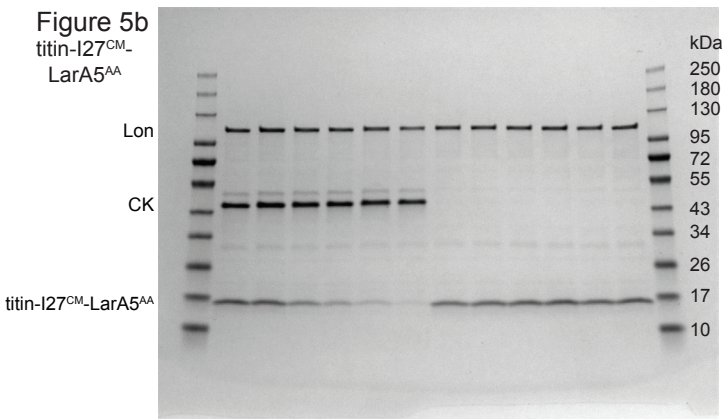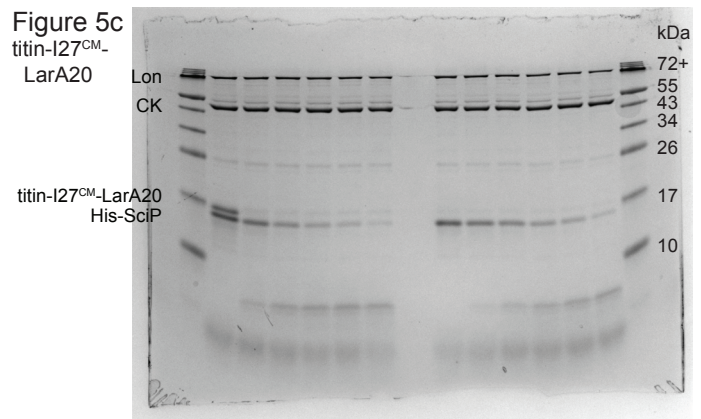

Supplement: Supplementary file 6 — Source Data [file 41467_2023_43385_MOESM6_ESM.zip › Figure 5 - Uncropped blots and gels.pdf]

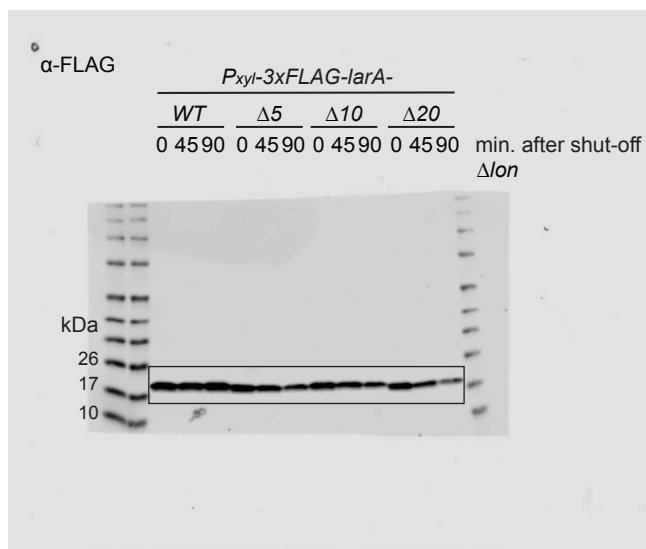

Supplement: Supplementary file 6 — Source Data [file 41467_2023_43385_MOESM6_ESM.zip › Supplementary Figure 6 - Uncropped blots and gels.pdf]

Supplementary Figure 8

Supplementary Figure 8a  
 $\alpha$ -LarA

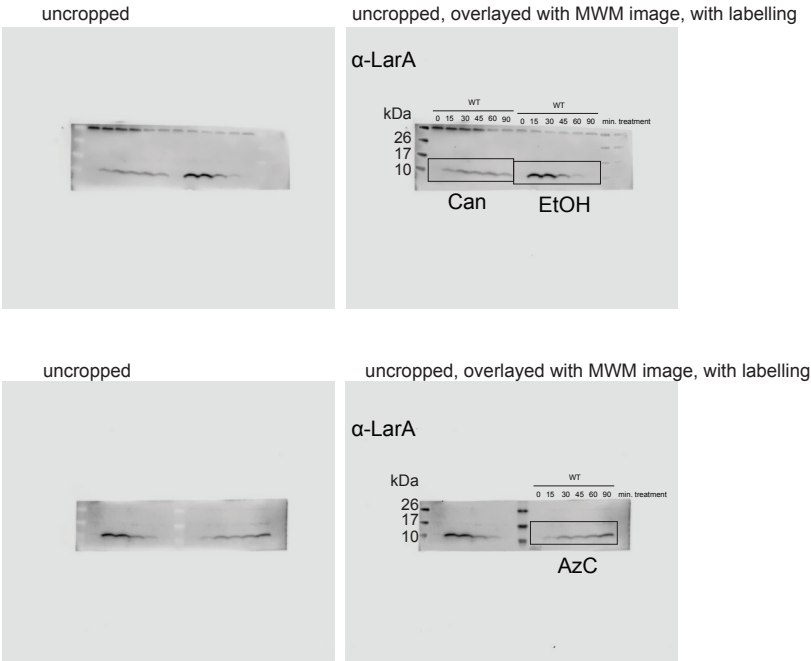

Supplement: Supplementary file 6 — Source Data [file 41467_2023_43385_MOESM6_ESM.zip › Supplementary Figure 8 - Uncropped blots and gels.pdf]
